# Osteopontin alters DNA methylation through up-regulating DNMT1 and sensitizes CD133+/CD44+ cancer stem cells to 5 azacytidine in hepatocellular carcinoma

**DOI:** 10.1186/s13046-018-0832-1

**Published:** 2018-07-31

**Authors:** Xiaomei Gao, Yuanyuan Sheng, Jing Yang, Chaoqun Wang, Rui Zhang, Ying Zhu, Ze Zhang, Kaili Zhang, Shican Yan, Haoting Sun, Jinwang Wei, Xuan Wang, Xinxin Yu, Yu Zhang, Qin Luo, Yan Zheng, Peng Qiao, Yue Zhao, Qiongzhu Dong, Lunxiu Qin

**Affiliations:** 1 Department of General Surgery, Huashan Hospital and Cancer Metastasis Institute and Institutes of Biomedical Sciences, Fudan University, Shanghai, 200032 China; 2 Department of General, Visceral and Cancer Surgery, University Hospital of Cologne, Cologne, Germany

**Keywords:** DNA methylation, Osteopontin, CD133+/CD44+ cells, Cancer therapy, Hepatocellular carcinoma

## Abstract

**Background:**

In hepatocellular carcinoma (HCC), CD133+/CD44+ cells are one subgroup with high stemness and responsible for metastatic relapse and resistance to treatment. Our previous studies have demonstrated that osteopontin (OPN) plays critical roles in HCC metastasis. We further investigated the molecular mechanism underlying the role of OPN in regulating the stemness of HCC epigenetically and explored possible targeting strategy.

**Methods:**

CD133+/CD44+ subgroup sorting from HCC cell lines and HCC tissues was used to investigate the effects of OPN knockdown on stemness. iTRAQ and MedIP-sequencing were applied to detect the protein profile and epigenetic modification of CD133+/CD44+ subgroup with or without OPN knockdown. The antitumor effects of 5 Azacytidine were examined in cultured HCC cells and patient derived xenograft (PDX) models.

**Results:**

OPN was accumulated in CD133+/CD44+ subgroup of HCC cells. Knocking down OPN significantly inhibited the sphere formation and stemness-related genes expression, and delayed tumor initiation of CD133+/CD44+ subgroup of HCC cells. Employing MedIP-sequencing, dot blot and iTRAQ analyses of CD133+/CD44+ SCR and CD133+/CD44+ shOPN cells, we found that OPN knockdown leaded to reduction in DNA methylation with particular enrichment in CGI. Meanwhile, DNA (cytosine-5)-methyltransferase 1 (DNMT1), the main methylation maintainer, was downregulated via proteomics analysis, which mediated OPN altering DNA methylation. Furthermore, DNMT1 upregulation could partially rescue the properties of CD133+/CD44+ shOPN cells. Both in vitro and in vivo assays showed that CD133+/CD44+ cells with high OPN levels were more sensitive to DNA methylation inhibitor, 5 Azacytidine (5 Aza). The above findings were validated in HCC primary cells, a more clinically relevant model.

**Conclusions:**

OPN induces methylome reprogramming to enhance the stemness of CD133+/CD44+ subgroup and provides the therapeutic benefits to DNMT1 targeting treatment in HCC.

**Electronic supplementary material:**

The online version of this article (10.1186/s13046-018-0832-1) contains supplementary material, which is available to authorized users.

## Background

Metastasis is a major hallmark of cancer, and the major death cause of cancer patients [1]. Many studies indicate that tumor heterogeneity is responsible for tumor metastasis, relapse and drug resistance of cancers including hepatocellular carcinoma (HCC) [2]. Cancer stem cells are conceited as the cells within the tumors possessing the capacities of self-renewal and heterogeneous lineages differentiation [3]. HCC cells sorted by CD133 [4] and CD44 [5] were regarded as a subpopulation of cells with stem cell properties [6]. It is convinced that specifically targeting cancer stem-cells is a promising approach for cancer treatment based on a detailed understanding of this unique sub-group biological features distinct from its parental counterparts [7]. Abnormally activated signal pathways and proteins in this subgroup could all be potential therapeutic targets [8, 9].

Stroma factors from tumor cells or stromal cells are critical for tumor metastasis [10]. Our previous studies have demonstrated OPN is a leading gene that promotes HCC metastasis [11, 12]. Intracellular/nuclear OPN regulated epithelial-mesenchymal plasticity, which contributes to the increased population of cancer stem-like cells (CSCs), to enhance tumor metastasis [13, 14]. In addition, OPN and its various cleavages in the tumor microenvironment serve as hematopoietic stem cell niche components that negatively regulates the anchorage and pool size of stem cells [15, 16]. OPN is also highly expressed in cancer stem cells isolated from HCC cell lines [17], and secreted OPN-CD44 signaling enhanced the phenotypes of cancer stem cells in glioma and colon cancer and promoted their aggressive tumor growth [18, 19]. These indicate OPN contributes to a cancer stem-like phenotype.

Epigenetic regulation has an important contribution in the development of adult stem cells, and its important roles in silencing tumor repressors and differentiation-associated genes in precancerous cells have drawn much attention recently [20]. Aberrant DNA methylation, the best studied epigenetic modification, has emerged as promising therapeutic targets to treat cancer. In ovarian cancer, epigenetic targeting agent can reprogram residual cancer stem-like cells [21]. DNMT1 is the most abundant type of DNMTs which catalyzes DNA methylation. In tumor tissues or cancer stem cells, DNMT1 is highly up-regulated [22, 23], and is required for maintaining the state of cancer stem cells [24]. DNMT1 inhibitor, 5 Aza, an FDA-approved drug to treat myelodysplastic syndromes (MDS), can eradicate cancer stem cells of solid tumor by inducing cell apoptosis or differentiation [25].

In the present study, we found that knockdown of OPN in CD133+/CD44+ cells inhibited sphere formation and migration through modulating the expression of DNMT1. Decreased levels of DNMT1 by OPN knockdown leaded to reduction in global DNA methylation, particularly in CpG island (CGI). In addition, CD133+/CD44+ subgroup from cell lines and HCC tissues with various OPN levels showed different sensitivities to 5 Aza. These results provide a potential significance of effective specific target therapy that epigenetic treatment is more effective for HCCs with high OPN expression.

## Materials and methods

### Magnetic activated cell sorting (MACS)

1 × 10^7^ cells were prepared and incubated with CD133 microbeads for 30 min. 1-2 mL MACS running buffer was added and the sample was centrifugated at 2000 rpm, 3 min and resuspended in 500ul MACS running buffer. The cells were placed in the prepared LS column and the column was washed for 6 times. Cells were removed from the column with physical forces. Then the single positive cells were incubated with CD44 microbeads and repeat the steps as before.

Immunohistochemical staining was performed according to the previous work [11].

### Cell culture and primary cells isolation

Huh7 and Hep3B were purchased from Chinese Academy, Shanghai and cultured in Dulbecco’s modified Eagle’s medium (DMEM) (Hyclone) with 10% FBS (Biowest). Primary cells were isolated from HCC tissues (from Huashan Hospital, Fudan University, Shanghai) by using IV type collagenase (Sigma) digested for 30 min and centrifugation for 1 min at 50 g.

### Patients and clinical tissue samples

A total of 374 patients who underwent curative resection for HCC at the authors’ institute between 2005 and 2006, were enrolled in the present study. None of them received any preoperative adjuvant treatment. The clinical samples were collected from patients after obtaining informed consent in accordance with an established protocol approved by the Ethics Committee of Fudan University (Shanghai, China).

### Spheroid-based migration assay on matrix protein

Flat-bottomed, 96-well plates (Corning) were coated with 0.1% gelatin (Sigma) in sterile water for 30 min at 37 °C. After removing the gelatin, 4-day spheroid was transferred into the plate cultured with 200ul/well medium supplemented with 2% FBS. Spheroids could adhere and migrate. Images were acquired at 0 h and 48 h using a stereomicroscope (Olympus, Shinjuku-ku, Tokyo, Japan) and analyzed by contrasting the area covered by migrating cells [26].

### iTRAQ assay

iTRAQ-8plex labeling reagents (AB Sciex) were added to the peptide samples. And then the peptides were fractionated on a waters UPLC using a C18 column. The fraction was separated by Nano-HPLC (Eksigent Technologies) on the secondary RP analytical column (Eksigent, C18, 3 μm, 150mmx75μm). Peptides were subsequently analyzed by the mass spectrometer (QTOF 5600). For the data processing of iTRAQ experiments, protein identification and iTRAQ 8 plex quantification were performed with ProteinPilot4.5 software (AB Sciex).

### MeDIP-sequencing

Genomic DNA of cells was purified, sonicated and then denatured. Denatured DNA was incubated with 5mC antibody (Active Motif) at 4 °C overnight. DNA-antibody complexes were captured by protein A/G beads (Santa Cruze). Harvested DNA was purified and sequenced followed by standard Illumina protocols. Read sequences were mapped to the human genome (hg38) using ELAND v2 in the CASAVA (Illumina, v1.6) package.

### LC-MS/MS analysis

LC-MS/MS analysis Genomic DNA (8 μg) from cultured cells was digested with DNA Degradase Plus (Zymo Research) at 37 °C for 3 h. The dideoxycytidine (TCI) was added as an internal control. The digested samples were then subjected to LC-MS/MS analysis using a ShimazuLC (LC-20AB pump) system coupled with TSQ-Vantage triple quadrupole mass spectrometer (Thermo). A C18 column (250 mm × 2.1 mm I.D., 3 μm particle size, ULTIMATE) was used. The mass spectrometer was optimized and set up in selected reaction monitoring (SRM) scan mode for monitoring the [M + H+] of 5-mC (m/z 242.1 → 126.1), 5-hmC (258.1 → 142.1), 5-fC (256.1 → 140.1), 5-caC (272.1 → 156.1) and dideoxycytidine (212.1 → 112.1). The Analyst Software was used for analysis.

### Xenograft assay

For detection of the ability of tumor initiation, diluted cells mixed with matrigel were subcutaneously implanted into the 4–6 weeks-old NOD SCID mice. Tumor formation was monitored each week. To evaluate the rate of inhibition of 5 Aza, 30 mg/kg 5 Aza was intraperitoneally injected into the nude mice when they burdened a tumor in almost the same volume for five times a week.

### Methylated DNA immunoprecipitation sequencing data analysis

Sequencing adapters were removed and low-quality bases (quality < 20) were trimmed from the 5′ and 3′ ends of reads using an in-house Perl script. The obtained clean reads were then mapped to the human reference genome (hg38) using the default parameters of the BWA program (version 0.7.7). Peaks of MeDIP-seq were identified by MACS2, then we merge the peak of CSCs-SCR and CSCs-shOPN samples. Static the fragments of each samples in enriched regions. Then we identified differentially methylated regions refer to a previously published method of differentially expressed genes [27]. *p* values were adjusted by false discovery rate (FDR) for multiple tests. A threshold of FDR < 0.05 and fold change > 2 was applied.

### Statistics analysis

All data are expressed as the mean ± standard deviation. Error bars represent ± standard deviation for triplicate experiments. The difference between groups was analyzed using Student *t*-test when comparing only two groups or one-way analysis of variance when comparing more than two groups. The level of significance was set at *p* < 0.05(*p < 0.05, **p < 0.01, ***p < 0.001).

**Plasmids construction and transfection**

The plasmids of OPN and DNMT1 were constructed as described in the Additional file 1.

**Sphere formation**

Sphere formation assay was assessed as detailed in the Additional file 1.

**RNA isolation, Reverse-transcription, and quantitative PCR analysis**

Quantitative PCR analysis (qPCR) were carried out as detailed in the Additional file 1.

**Western blot assay**

Whole cell lysis was produced by using RIPA buffer containing protease inhibitor. Western blot was performed as described in the Additional file 1.

**Flow cytometry**

Flow cytometry analysis was performed as described in the Additional file 1.

**Dot blot assay**

Genomic DNA was extracted by using phenol-chloroform and diluted into the same concentration. The detailed method was described in the Additional file 1.

**Methylation-Specific PCR, MSP**

Genomic DNA was isolated from the cells. MSP assay was done as described in the Additional file 1.

## Results

### OPN enhances the stemness of CD133+/CD44+ subgroup

**Fig. 1:**
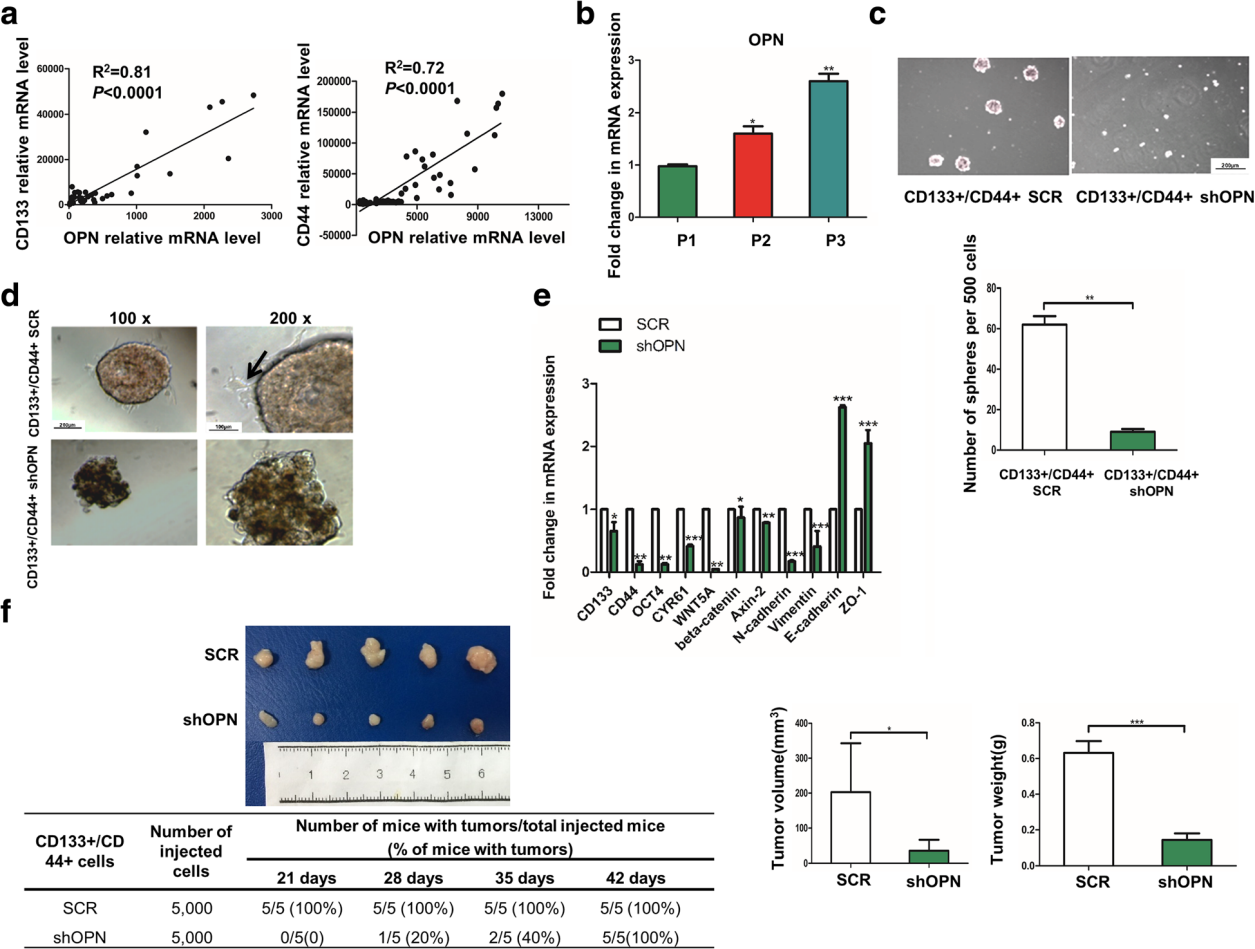
OPN regulates the properties of CD133+/CD44+ cells. **a** OPN was tightly correlated with the expression of CD133 and CD44 in the clinical samples (Data from TCGA). **b** The expression level of OPN during generation passaging. **c** OPN knockdown in CD133+/CD44+ cells decreased the number of sphere formation significantly, 100×, **, *p* < 0.01. **d** Knockdown of OPN impeded the migration on gelatin of CD133+/CD44+ cells. **e** Stemness-related genes were down-regulated by comparing with CD133+/CD44+ SCR and CD133+/CD44+ shOPN. **f** In vivo, 5000 cells of CD133+/CD44+ SCR and CD133+/CD44+ shOPN were subcutaneously implanted, shOPN delayed the emergency and slowed down the increase rate of tumor, *, *p* < 0.05 and ***, *p* < 0.001

CD133+/CD44+ cells had been approved to have stem/progenitor cell properties in HCC. Our previous studies have demonstrated that OPN is one of the leading genes that promote HCC metastasis [11, 12, 28]. By analyzing the data from The Cancer Genome Atlas (TCGA) database, OPN was found to be significantly correlated with the expression of CD133 and CD44 (Fig. 1a). Therefore, we focused on the function and regulation mechanism of OPN in CD133+/CD44+ subgroup of HCC.

We sorted CD133+/CD44+ cells from Huh7 by magnetic activated cell sorting (MACS). In this subgroup, the level of OPN was accumulated during generation passaging (Fig. 1b). Knockdown of OPN by shRNA (shOPN) significantly suppressed the abilities of sphere formation and migration of CD133+/CD44+ cells (Additional file 2: Figure S1, Fig. 1c, d), and down-regulated the key genes related to the pluripotency (OCT4, Nanog), stemness (CD133, CD44, CYP61) and EMT (beta-Catenin, Vimentin, N-cadherin, E-cadherin, ZO-1) (Fig. 1e). And it also dramatically delayed the in vivo tumor initiation and diminished the tumor sizes of CD133/CD44+ cells from Huh7 (Fig. 1f). Moreover, putting-back OPN recovered the sphere formation ability and the expression levels of the down-regulated genes in CD133+/CD44+ cells with shOPN separated from Huh7 (Additional file 2: Figure S2A, B). Comparable results were also observed in CD133+/CD44+ cells from Hep3B (Additional file 2: Figure S2 C, D, E, F, G and H).

In contrast, the sphere formation potential and the expression levels of the stemness-related genes were significantly elevated in CD133+/CD44+ subgroup from Huh7 with over expressed OPN (Additional file 2: Figure S3 A, B and C). And both the in vivo tumor formation latency and tumor size were closely correlated with OPN level of CD133+/CD44+ cells. Remarkably, CD133+/CD44+ cells with high-OPN generated tumors earlier and their tumor sizes were larger than the controls (Additional file 2: Figure S3 D). All these findings demonstrate that OPN plays important roles in enhancing the capacities of CD133+/CD44+ cells in HCC.

### OPN knockdown reduces DNA methylation in CD133+/CD44+ subgroup

**Fig. 2:**
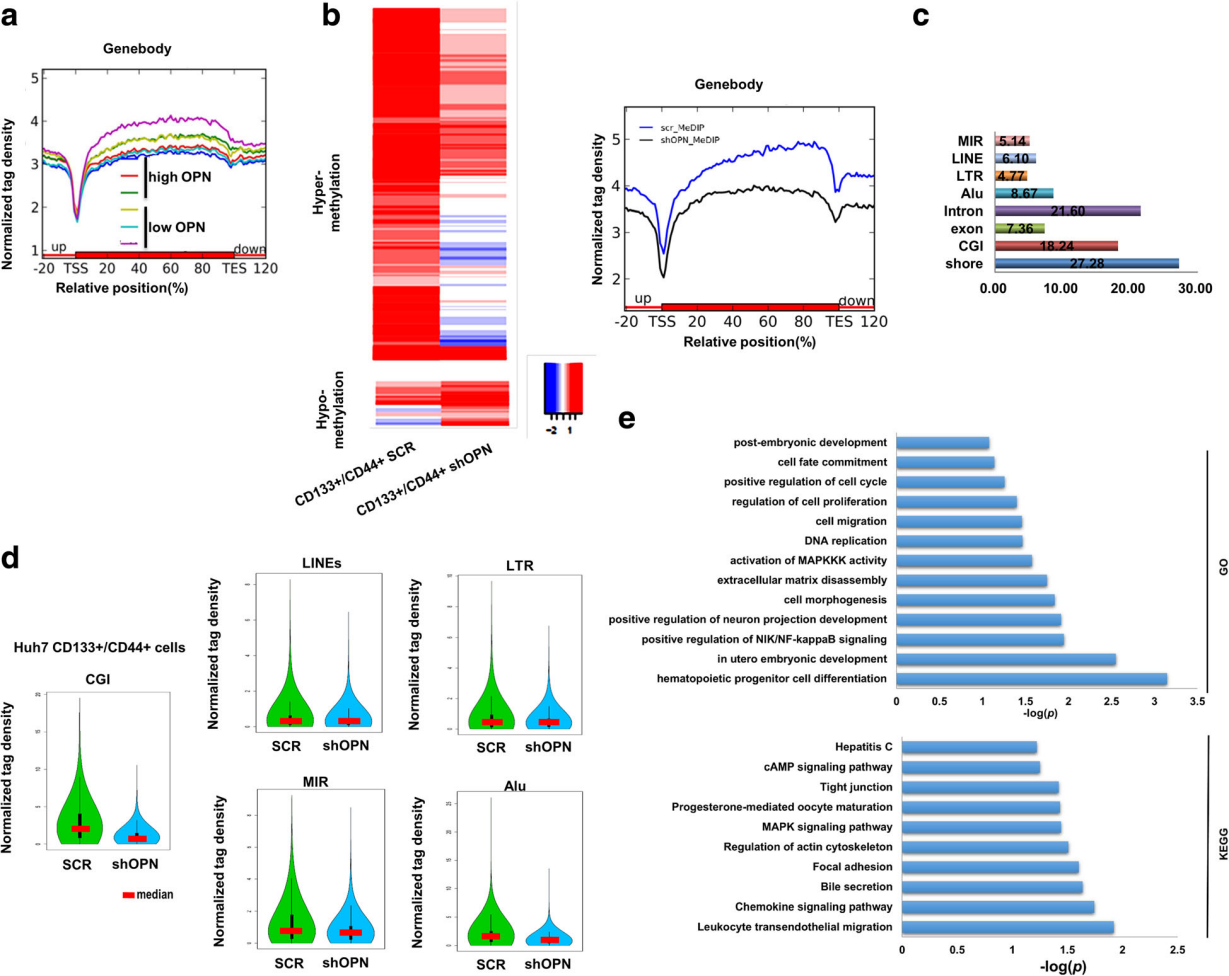
MeDIP-seq analysis of OPN knockdown in CD133+/CD44+ cells. **a** Normalized 5mC tag density distribution across the genebody. Each gene was normalized to 0–100%. **b** The heatmap of differentially methylated tiles (left) and normalized 5mC tag density distribution (right) in CD133+/CD44+ cells with SCR or shOPN from Huh7 using MeDIP-seq. **c** Average percentile changes in methylation in the different elements. **d** Distribution of methylation density within retrotransposon elements. Red lines presented median values. **e** KEGG and GO analyses of MeDIP-seq in CD133+/CD44+ cells with SCR or shOPN

Many evidences show that OPN genetically enhances tumor growth and metastasis in HCC. Whether OPN had epigenetic function was still unknown. To examine if OPN was involved in the regulation of DNA methylation, we performed MeDIP-sequencing (MeDIP-seq) assay in HCC tissues, and found an increasing tendency in 5-methylcytosine (5mC) differentially modified peaks in HCCs with high OPN expression than that of low-OPN ones (Fig. 2a). And in CD133+/CD44+ cells, we also mapped genomic methylation changes by conducting MeDIP-seq of CD133+/CD44+ SCR and CD133+/CD44+ shOPN, and found a significantly decreased global DNA methylation in CD133+/CD44+ cells with shOPN compared with that of the control group (Fig. 2b). To further analyze MeDIP-seq data sets, the distribution of differentially methylated regions (DMRs) were mostly enriched in CpG island (CGI) rather than the retrotransposon elements (Fig. 2c). The CGIs comprise a substantial proportion of the genome and function as important modulators of host gene expression [29]. Detailed analysis on DMRs in CGIs revealed that knockdown of OPN in CD133+/CD44+ cells resulted in a distinct density pattern (Fig. 2d). With KEGG and GO analyses, differentially methylated genes were enriched in some key signaling pathways (Fig. 2e).

**Fig. 3:**
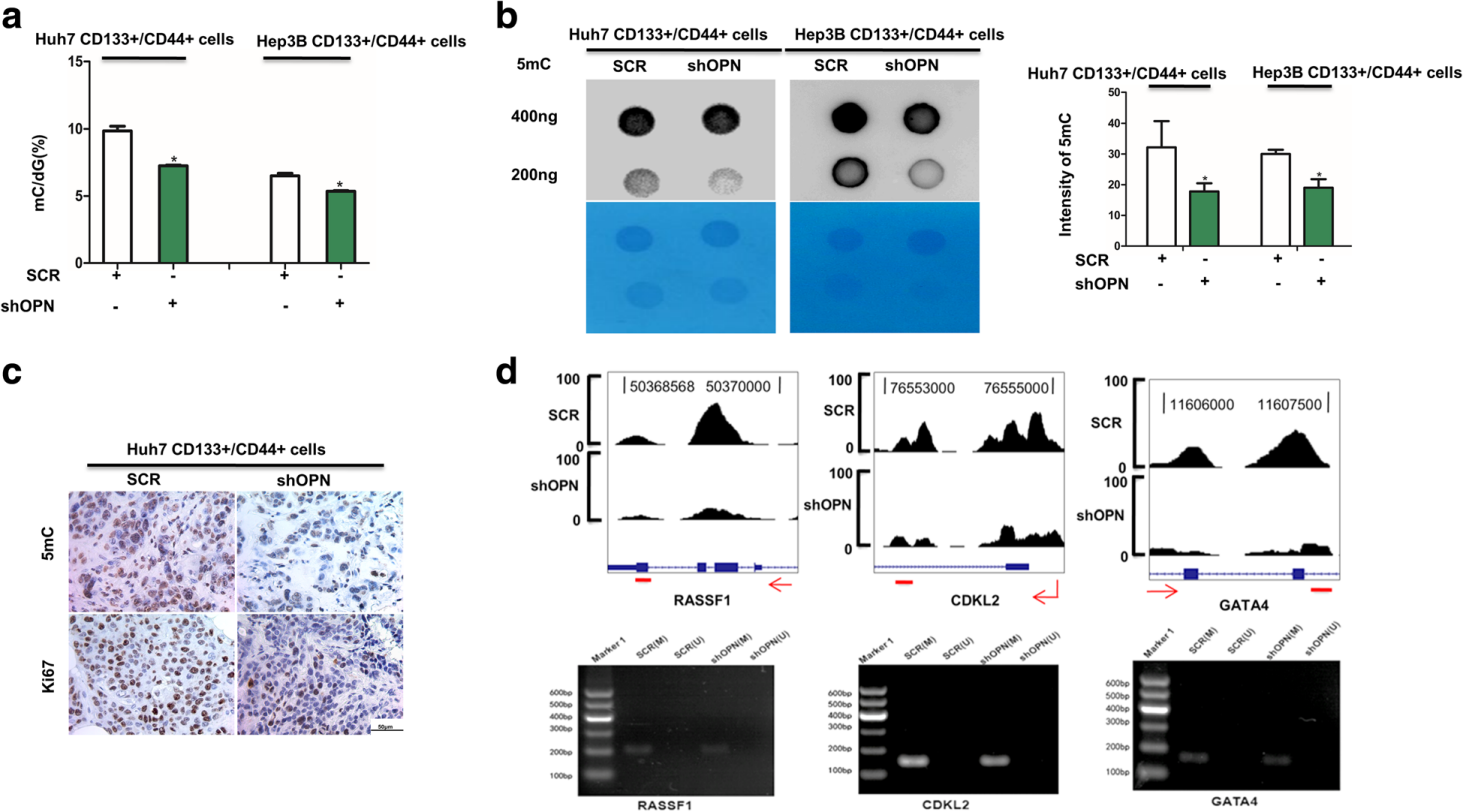
OPN alters DNA methylation in CD133+/CD44+ cells. **a** The ratio of mC in total cytosine in CD133+/CD44+ cells with SCR or shOPN from Huh7 and Hep3B, *, *p* < 0.05. **b** Dot blot of DNA isolated from CD133+/CD44+ cells with SCR or shOPN from Huh7 and Hep3B probed with anti-5mC antibody. Quantified signal was normalized to total DNA as measured by methylene blue staining, *, *p* < 0.05. **c** IHC staining for 5mC and Ki67 in the tumor tissues formed by CD133+/CD44+ cells with SCR or shOPN from Huh7. **d** MeDIP-seq results of *RASSF1*, *CDKL2* and *GATA4* genes (up) and confirmation by MSP-PCR (low)

This was confirmed by dot blot assay and LC-MS/MS assay in CD133+/CD44+ subgroup isolated from Huh7 and Hep3B cells (Fig. 3a, b). In addition, we investigated DNA methylation in subcutaneous models of CD133+/CD44+ SCR and CD133+/CD44+ shOPN. Immunohistochemical (IHC) staining demonstrated that 5-methylcytosine (5mC) was down-regulated in subcutaneous tumor tissues from implantation models of CD133+/CD44+ shOPN compared with CD133+/CD44+ SCR. Accordingly, staining intensities for Ki67 were greatly decreased in tumor tissues from mice with subcutaneous HCC implantation of CD133+/CD44+ shOPN cells (Fig. 3c). *RASSF1*, *GATA4* and *CDKL2* were examples of differentially methylated genes (Additional file 2: Figure S4). OPN knockdown reduced methylation of these three genes using methylation-specific PCR (MSP) (Fig. 3d).

These data further support that OPN induces aberrations in genomic methylation of CD133+/CD44+ cells in HCC.

### DNMT1 mediates OPN altering DNA methylation in CD133+/CD44+ subgroup

**Fig. 4:**
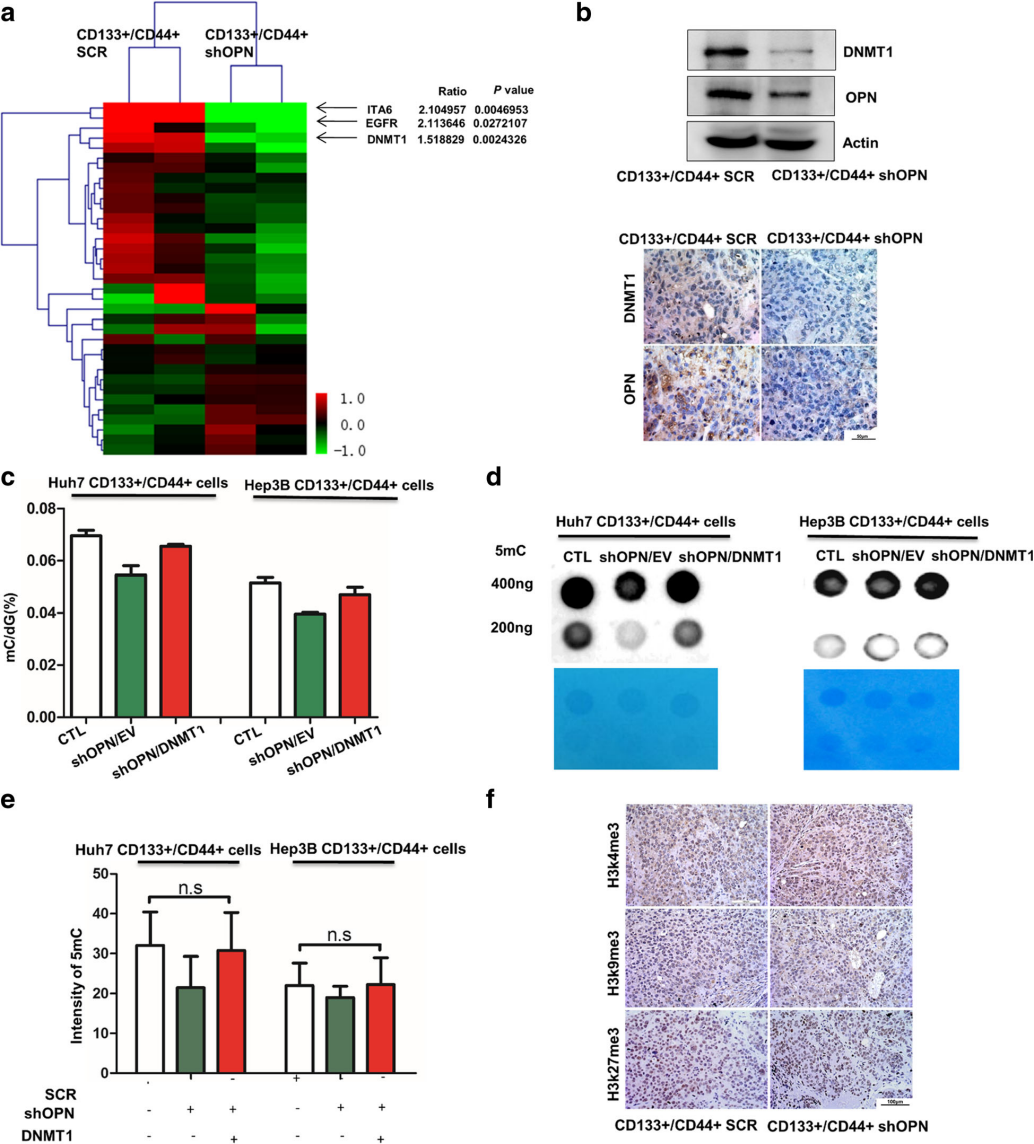
DNMT1 mediates OPN altering DNA methylation in CD133+/CD44+ cells. **a** Compared the protein file of CD133+/CD44+ SCR with that of CD133+/CD44+ shOPN sorted from Huh7, the top different proteins were picked up. **b** Using IHC and western blot, DNMT1 was down-regulated when knocking down OPN. **c** The ratio of mC in total cytosine in CD133+/CD44+ cells with SCR or shOPN/EV or shOPN/DNMT1 from Huh7 and Hep3B, *, *p* < 0.05, ns, no significance. **d** Dot blot of DNA isolated from CD133+/CD44+ cells with SCR or shOPN/EV or shOPN/DNMT1 from Huh7 and Hep3B probed with anti-5mC antibody. **e** Quantified signal was normalized to total DNA as measured by methylene blue staining, ns, no significance. **f** IHC analysis of histone 3 methyl marks, H3K4me3, H3K9me3 and H3K27me3 in the tumor tissues formed by CD133+/CD44+ cells with SCR or shOPN from Huh7

To elucidate the detailed molecular mechanisms of OPN in modulating DNA methylation, the proteome profiles of CD133+/CD44+ cells with shOPN and the control group were constructed by iTRAQ assay. In agreement with our observation in HCC tissues, ITA6 and EGFR were found to be significantly decreased in CD133+/CD44+ cells with shOPN (Fig. 4a). After statistical analysis, differentially expressed proteins were almost enriched to cellular growth and proliferation consistent with previous studies (Additional file 2: Figure S5A). Besides, some signaling pathways related to chromosome stability and regulating gene expression epigenetically were also enriched (Additional file 2: Figure S5B). Noticeably, DNMT1, an essential regulator maintaining both epigenetic reprogramming during DNA replication and genome stability, was identified by this mass spectrum data with an average of 1.5-fold down-expression in CD133+/CD44+ shOPN (Fig. 4a). Immunoblot and IHC results showed the expression of DNMT1 was decreased by OPN knockdown in CD133+/CD44+ cells (Fig. 4b). To confirm the alteration of genomic methylation caused by OPN-DNMT1 axis, DNMT1 over expression plasmid was induced into CD133+/CD44+ cells with shOPN from Huh7 and Hep3B. With dot blot assays and LC-MS/MS analysis, the effect of shOPN on DNA methylation was reversed by DNMT1 over expression in CD133+/CD44+ subgroup. (Fig. 4c-e).

Meanwhile, there was no significant change in expression levels of H3K4me3, H3K9me3 and H3K27me3, which excluded the function of histone methylation on gene expression (Fig. 4f). That means OPN mainly induced aberrant DNA methylation by regulating DNMT1 expression.

### OPN knockdown decreases the stemness of CD133+/CD44+ subgroup through down-regulating DNMT1

**Fig. 5:**
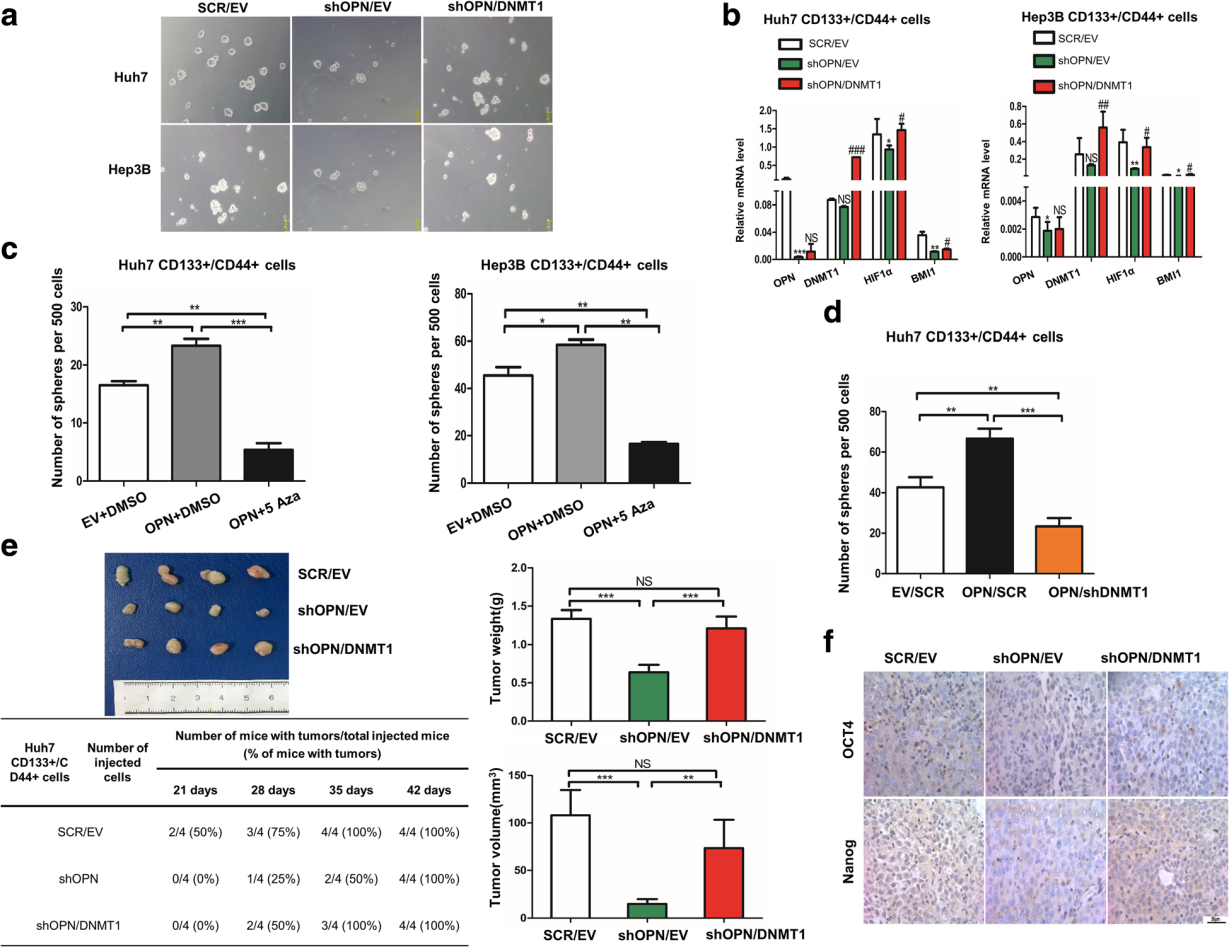
OPN modulates the properties of CD133+/CD44+ cells through regulating DNMT1. **a** When CD133+/CD44+ cells with shOPN was transfected with DNMT1 from Huh7 and Hep3B, the ability of sphere forming was rescued compared to the shOPN group, 40X. **b** The expression of indicated genes down regulated in CD133+/CD44+ cells from Huh7 and Hep3B transfected with shOPN compared to the control, were reversed in part by DNMT1 transfection. **c** The number of spheres formed by CD133+/CD44+ cells with EV, CD133+/CD44+ cells with OPN and CD133+/CD44+ cells with OPN treated by 5 Aza, **, *p* < 0.01, ***, *p* < 0.001. **d** The number of spheres formed by CD133+/CD44+ cells with EV/SCR, CD133+/CD44+ cells with OPN/SCR and CD133+/CD44+ cells with OPN/shDNMT1, **, *p* < 0.01. **e** CD133+/CD44+ SCR/EV, CD133+/CD44+ shOPN/EV and CD133+/CD44+ cells shOPN/DNMT1 from Huh7 were injected subcutaneously into the mice, and recorded the time of tumor initiation, tumor weight and tumor volume, **, *p* < 0.01, ***, *p* < 0.001, NS, no significance. **f** IHC staining of stemness-related markers, OCT4 and Nanog in the tumor tissues derived from CD133+/CD44+ cells with SCR/EV or shOPN/EV or shOPN/DNMT1

As DNMT1 is essential for tumorigenesis and stemness maintenance, we postulated that OPN might modulates the properties of CD133+/CD44+ cells through DNMT1. The potential of sphere formation could be rescued by introduction of DNMT1 in CD133+/CD44+ cells with shOPN (Fig. 5a, Additional file 2: Figure S6A). In addition, the downstream of DNMT1 including E-cadherin and GATA4 were significantly changed after knocking down OPN in CD133+/CD44+ cells sorted from Huh7 (Additional file 2: Figure S7 A, B). And as the key downstream effectors of OPN for cancer stemness [30, 31], the expression levels of BMI1 and HIF1α were increased when DNMT1 was up-regulated in CD133+/CD44+ cells with shOPN (Fig. 5b). To further confirm these findings, we treated OPN-up-regulated CD133+/CD44+ cells with 5 Aza and found it could neutralize the increased sphere formation of CD133+/CD44+ cells induced by OPN over expression (Fig. 5c), as did by blockade of DNMT1 (Fig. 5d).

**Fig. 6:**
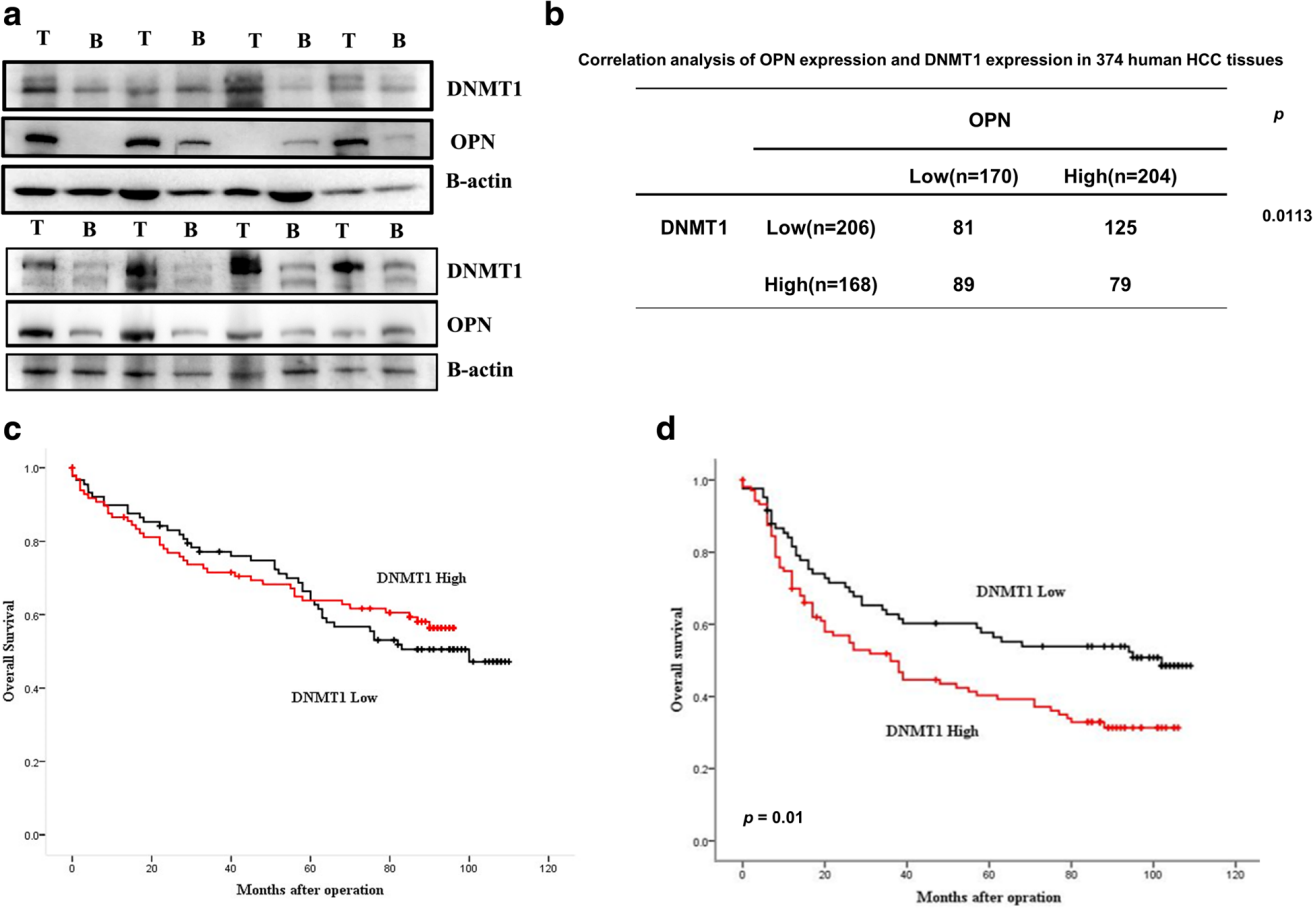
Prognostic significance of OPN and DNMT1 in HCC tissues. **a** Western blot analyses of OPN and DNMT1 in the HCC tissues. **b** The association between the expression of OPN and DNMT1 in HCC tissues. **c** Kaplan-Meier survival analysis of HCC tissues according to high OPN and high or low DNMT1 expression levels

To further determine the in vivo effect of DNMT1 on the increased stemness of OPN-over expressed HCC, CD133+/CD44+ cells with SCR/EV, CD133+/CD44+ cells with shOPN/EV and CD133+/CD44+ cells with shOPN/DNMT1 were subcutaneously implanted into NOD SCID mice. No significant difference was found in tumor initiation and the formed tumor sizes between CD133+/CD44+ cells with shOPN/DNMT1 and those with SCR/EV (Fig. 5e). And the expression of stemness markers, OCT4 and Nanog, which were down-regulated in tumor tissues from the subcutaneous implantation models of CD133+/CD44+ cells with shOPN/EV, were partly rescued by introduction of DNMT1 (Fig. 5f). Moreover, it is very interesting that OPN related tightly to DNMT1 only in the tumor tissue with grade four and macro vascular invasion in TCGA database (Additional file 2: Figure S7C). Further, we evaluated the association between OPN and DNMT1 in HCC tissues. Western blot and IHC showed that OPN expression was positively correlated with DNMT1 expression (Fig. 6a, b). Based on the expression of OPN and DNMT1, Kaplan-Meier analysis showed that patients with low OPN and high or low DNMT1 expression had no difference in prognosis (Fig. 6c). But patients with both high OPN and high DNMT1 expression had the shorter overall survival times (OS) than those with high OPN and low DNMT1 (Fig. 6d). Collectively, these data suggest that the OPN-DNMT1 signaling pathway promoted HCC stemness.

### HCCs with high OPN are more sensitive to DNMT1 inhibitor, 5 Aza

**Fig. 7:**
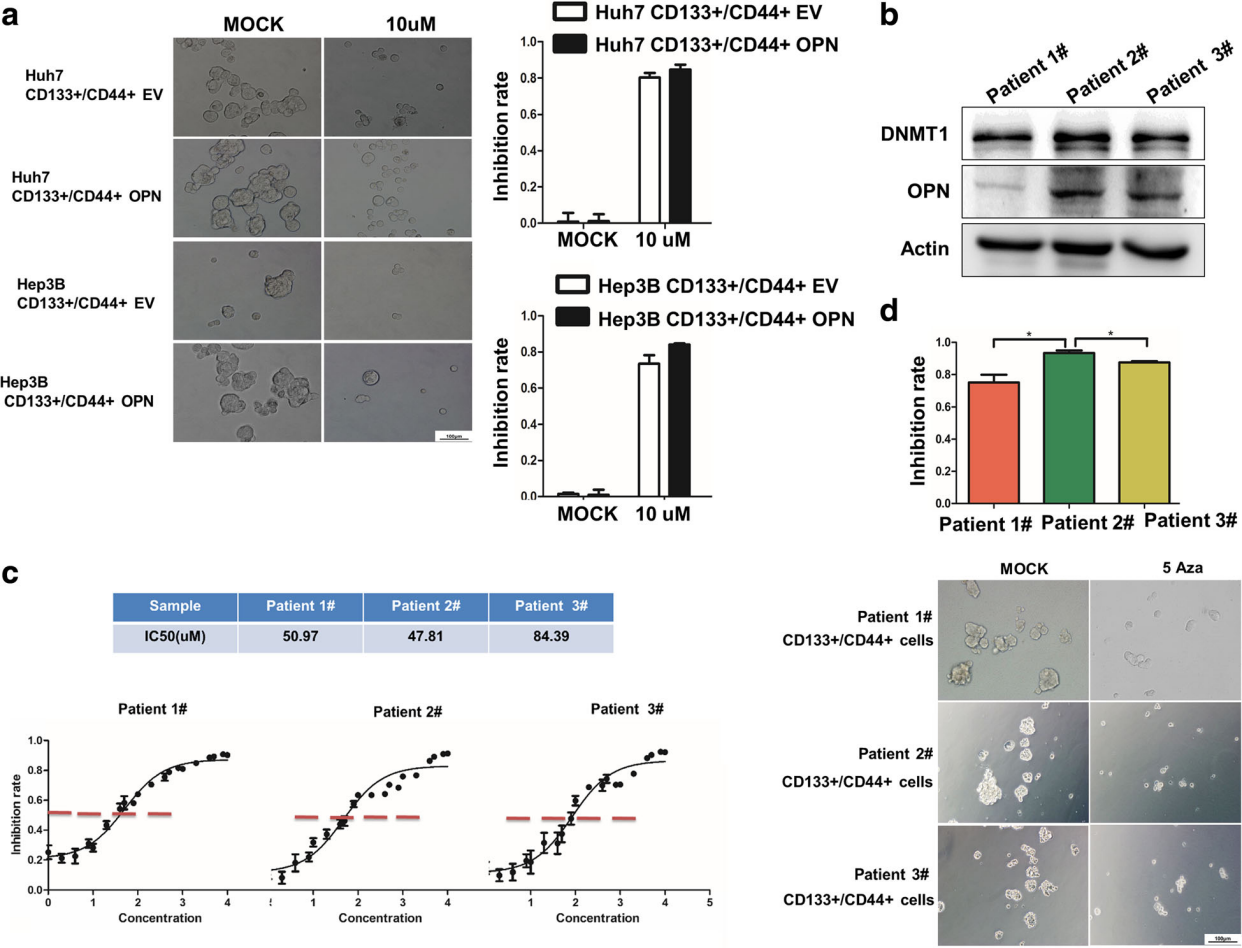
HCCs with high OPN are more sensitive to DNMT1 inhibitor, 5 Aza, in vitro. **a** The inhibition rate of 5 Aza in CD133+/CD44+ cells with EV or OPN from Hep3B and Huh7, 200×. **b** Immunoblot analysis of DNMT1 and OPN in the lysates from primary HCC cells. **c** IC50 values of three primary HCC cells. **d** The inhibition rate of 5 Aza in CD133+/CD44+ cells from primary HCC cells, 200×, *, *p* < 0.05

**Fig. 8:**
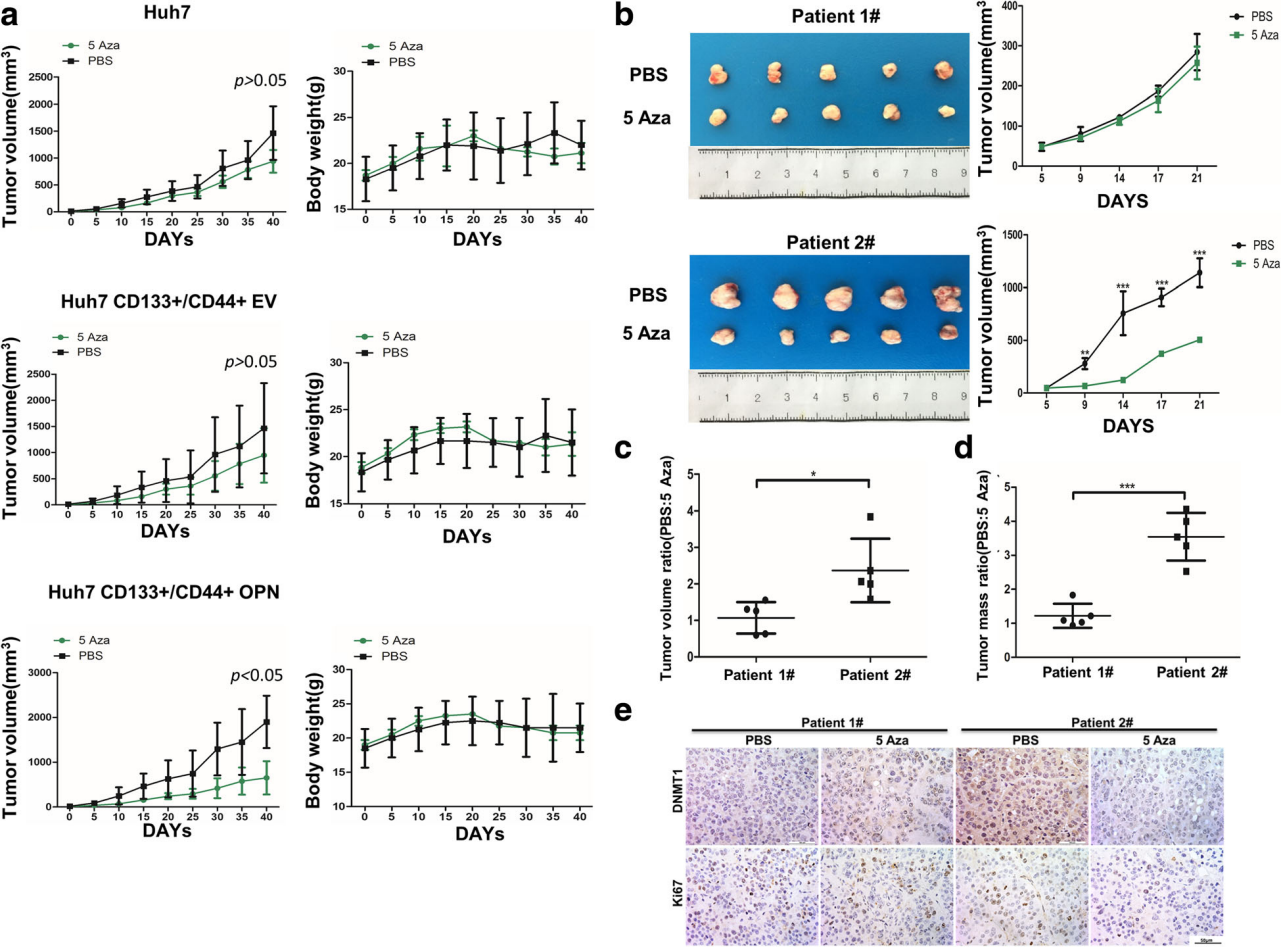
HCCs with high OPN are more sensitive to DNMT1 inhibitor, 5 Aza, in vivo. **a** Volume of subcutaneous tumors derived from Huh7 cells and CD133+/CD44+ cells with EV or OPN isolated from Huh7 treated with PBS or 5 Aza (left), and body weight of mice with subcutaneous implantation of Huh7 cells and CD133+/CD44+ cells with EV or OPN (right). **b** Two HCC primary xenograft models treated with PBS or 5 Aza. Tumor size and volume between the PBS-treated and 5 Aza-treated tumors, **, *p* < 0.01, ***, *p* < 0.001. **c**-**d** A mean tumor volume ratio (PBS:5 Aza) and a mean tumor mass ratio (PBS:5 Aza) were seen in PDTX, *, *p* < 0.05, ***, *p* < 0.001. (E) IHC staining of DNMT1 and Ki67 in the subcutaneous tumor tissues from HCC primary xenograft models treated with PBS or 5 Aza

5 Aza, a specific inhibitor of DNMT1, can lead to a reduction in DNA methylation and activation of transcriptionally silenced genes [32]. We sought to evaluate the functional implications of OPN expression in tumor sensitivity towards 5 Aza treatment of HCCs. 5 Aza treatment, even at a minimal dosage, could markedly suppress the sphere formation of CD133+/CD44+ cells with up-regulated OPN (Fig. 7a), and knockdown of OPN significantly diminished the sensitivity of CD133+/CD44+ cells to 5 Aza treatment (Additional file 2: Figure S8A). In addition, we further evaluated the effect of 5Aza treatment in three primary cell lines derived from HCC tissues with high or low OPN (Fig. 7b). CD133+/CD44+ cells from high-OPN tissues were more sensitive to 5 Aza than the other two with lower OPN (Fig. 7c, d). Moreover, in subcutaneous implantation nude mice models, 5 Aza induced a higher inhibition rate on tumor growth of CD133+/CD44+ cells with high-OPN compared with the low-OPN controls (Fig. 8a). These indicate that OPN is closely related to the sensitivity of CD133+/CD44+ cells to 5Aza.

To further confirm that DNMT1 inhibitor could offer therapeutic benefit to HCC patients with high OPN, we established two HCC primary xenograft (PDX) models. Tissue sections from these models were characterized using IHC analysis for OPN (Additional file 2: Figure S8B). They were subjected to the same 5 Aza treatment as subcutaneous implantation models. PDTX#2 from high OPN HCC showed a remarkable tumor-suppressive response to 5 Aza (Fig. 8b). In contrast, the tumor volume in PDTX#1 from low-OPN HCC was still increased moderately after 5 Aza treatment. Comparing with the PBS controls, in the 5 Aza-treatment group, the differences in both the mean tumor volume ratio (Fig. 8c) and mean tumor mass ratio (Fig. 8d) were greater in PDTX#2 than PDTX#1. And a greater reduction of Ki67 protein was found in PDTX#2 after 5 Aza treatment (Fig. 8e).

## Discussion

High probabilities of metastatic relapse and drug resistance remain the major obstacle to further prolong the survival of patients with HCC [33]. CD133+ and CD44+ were proposed to be markers of tumor-initiating cells (TICs) in liver cancers [34]. Targeting TICs may provide a practical approach for cancer treatment [35]. Many clinical trials and pre-clinical studies that specifically target TICs are under processing [36]. Acyclic retinoid (ACR), a selective chemopreventive agent, selectively cleared MYCN+ liver CSCs [37]. However, great breakthrough may be arduous to achieve for the dynamics and biomarkers uncertainty of HCC CSCs. Therefore, it is crucial to uncover the biology and phenotype regulation of CD133+/CD44+ subgroup in HCC.

OPN had been reported to be highly expressed in cancer stem cells and its secreted form regulated the self-renewal of cancer stem cells genetically. Cancer stem cells are able to undergo self-renewing division and differentiate into other kinds of cells [38]. With these properties of malignancy, cancer stem cells display phenotypical and functional heterogeneity accounting for therapeutic refractoriness and tumor dormancy [39]. Cancer stem cells were often distinguished and sorted by relevant specific biomarkers. The heterogeneity of CSCs is universal. CD44 has been reported to be marker of CSCs in many tumors. Ectopic expression of CD44v6 in colorectal CSCs by activating the Wnt/β-catenin pathway, promotes migration and metastasis. However, in gastric cancer, CD44v8–10 enriched at the invasive front attenuates redox-stress-induced canonical Wnt activation [40, 41]. The plasticity of CSCs depends on the cancer types and cell context. In this work, CD133+/CD44+ cells were approved to have the properties of cancer stem cells as reported. We found that OPN could enhance the self-renewal of CD133+/CD44+ cells via regulating DNMT1. Therefore, apart from the secreted form, intracellular OPN also contributes to the stemness of CD133+/CD44+ cells.

Aberrations in DNA methylation patterns contribute to cancer pathogenesis and malignancy, usually with a global DNA hypomethylation and local hypermethylation at specific tumor suppressor genes or differentiation-related genes. Abnormal DNA methylation at the 5 position of cytosine (5mC) is a well-known epigenetic feature of cancer [42]. Meanwhile, epigenetic transcriptional regulation is also crucial in the development and maintenance of cancer stem cells. However, whether OPN affected DNA methylation remained unknown. Using MeDIP-seq, we found that OPN altered DNA methylation landscape of CD133+/CD44+ cells via regulating the expression of DNMT1, a key enzyme of DNA methylation. With aberrant DNA hypermethylation due to low expression of DNMT1, the expressions of some tumor suppressors, involving genes aberrantly methylated in HCC and those as drivers of other cancers, were significantly up-regulated, which could break down the balance of self-renewal and differentiation of CD133+/CD44+ cells. Considering that epigenetic alteration precedes gene expression, OPN-induced differentially methylated genes are speculated to have strong potential as epigenetic biomarkers for cancer diagnosis, prognostication and therapeutic interventions. Collectively, these demonstrate that OPN induces genome-wide methylome alterations. This may be a mechanism exploited by OPN to enhance the stemness of CD133+/CD44+ cells via methylome reprogramming to antagonize apoptosis or differentiation.

When TICs are eradicated thoroughly by targeting the abnormally activated signaling pathways or protein, tumor can be possibly cured [43]. Targeting OPN can be a strategy for elimination of CD133+/CD44+ subgroup. Our lab has reported that neutralizing antibody to OPN can inhibit the in vitro invasion and in vivo lung metastasis of highly metastatic HCC cells. And microRNA against OPN led to an obvious inhibition of both in vitro invasion and in vivo lung metastasis of HCC-LM3 cells [11]. But OPN-specific inhibitory compounds are not available yet. Thus, there is no ideal targeting therapeutic strategy. We have to explore an alternative strategy that indirectly targets OPN by the signaling cascade components, leading to OPN-dependent HCC stemness. DNMT1 can be an ideal subrogation of targeting OPN in terms of the epigenetic therapeutic strategy. We found that CD133+/CD44+ cells with high OPN expression up-regulated DNMT1, which made CD133+/CD44+ cells more sensitive to DNMT1 inhibitor, 5Aza. This work also provides an evidence that 5 Aza can be used to treat CD133+/CD44+ cells from solid tumor and HCC cells with high OPN expression. However, DNMT1 has been reported to be with multiple functions. High DNMT1 was related with a dismal prognosis of HCC patients. However, some else reports demonstrated that low DNMT1 contributed to the stemness of cancer stem cell-like cells and treatment with 5 Aza in HCC cells could increase the number of cancer stem cell-like cells [44]. The real reason of the controversial results is not clear, and the possible mechanisms involved deserve further investigation. In a recent report, 5 Aza in combination with alendronate could reduce the dose of 5 Aza [45], and the combination of 5 Aza and HDAC inhibitors result in a better curative effect in mouse model. Our findings at least in part support that 5 Aza can be used to treat HCC, especially for those with OPN over-expression.

## Conclusions

Based on both in vitro and in vivo studies, OPN is proved to induce methylome reprogramming to enhance the stemness of CD133+/CD44+ cells and provides the therapeutic benefits to DNMT1 targeting treatment. Our findings reveal that OPN-DNMT1 axis plays important roles in the regulation of stemness through modulating DNA methylation of CD133+/CD44+ cells. These implicate OPN as a promising indicator for HCC treatment with 5 Aza.

## Additional files

Additional file 1: Supplemental materials and methods. (DOCX 16 kb)

Additional file 2: **Figure S1.** The expression of OPN and DNMT1. (A) qRT-PCR and immunoblot assay of OPN knockdown. (B) qRT-PCR assay of DNMT1 knockdown. **Figure S2.** OPN knockdown impaired the properties of CD133+/CD44+ cells. (A-B) OPN rescuing reversed the number of spheres and the expression of genes in Huh7, NS, no significance. (C) shOPN in CD133+/CD44+ cells from Hep3B reduced the number and size of spheres, 100x. (D) CD133 and CD44 were down-regulated in CD133+/CD44+ shOPN from Hep3B. (E) OPN knockdown in CD133+/CD44+ cells from Hep3B inhibited genes expression. (F) shOPN in Hep3B CD133+/CD44+ cells decreased the potential of migration on gelatin, 100x and 400x. (G-H) OPN rescuing reversed the number of spheres and the expression of genes in Hep3B. **Figure S3.** OPN strengthened the stemness of CD133+/CD44+ cells from Huh7. (A-C) OPN over-expression formed more spheres of larger size, 100x, and activated genes expression. (D) Mice injected with 1,000 cells of CD133+/CD44+ EV or OPN were monitored weight and volume of tumors. **Figure S4.** MeDIP-seq results of RASSF1, CDKL2 and GATA4. **Figure S5.** Statistical analysis of iTRAQ assay. (A) KEGG analyses in Huh7 CD133+/CD44+ cells with SCR or shOPN. (B) Signaling pathways analyses. **Figure S6.** DNMT1 rescued the potential of sphere formation of CD133+/CD44+ cells with shOPN. (A)The number of spheres formed by CD133+/CD44+ cells with SCR/EV, shOPN/EV or shOPN/DNMT1. **Figure S7.** OPN related to DNMT1 expression. (A) The expression of DNMT1-downstream genes in CSCs with SCR or shOPN. (B) Staining of E-cadherin and GATA4 in the tumor formed by CSCs with SCR or shOPN. (C) The correlation of OPN and DNMT1 in tumor tissues (data form TCGA). **Figure S8.** CD133+/CD44+ cells with low OPN showed less sensitivity to 5 Aza. (A) 5 Aza IC50 (μM) in CD133+/CD44+ cells with SCR or shOPN. (B) Staining of OPN in the patient tissues. (DOCX 2324 kb)

## Abbreviations

5 Aza: 5 Azacytidine
5mC 5: methylcytosine.
CGI: CpG island.
DNMT1: DNA (cytosine-5)-methyltransferase 1.
HCC: hepatocellular carcinoma.
HR: hazard ratio.
IHC: immunohistochemistry.
OPN: osteopontin.
SD: standard deviation.
