# Correction: Osteopontin alters DNA methylation through up-regulating DNMT1 and sensitizes CD133+/CD44 + cancer stem cells to 5 azacytidine in hepatocellular carcinoma

**DOI:** 10.1186/s13046-023-02852-5

**Published:** 2023-10-14

**Authors:** Xiaomei Gao, Yuanyuan Sheng, Jing Yang, Chaoqun Wang, Rui Zhang, Ying Zhu, Ze Zhang, Kaili Zhang, Shican Yan, Haoting Sun, Jinwang Wei, Xuan Wang, Xinxin Yu, Yu Zhang, Qin Luo, Yan Zheng, Peng Qiao, Yue Zhao, Qiongzhu Dong, Lunxiu Qin

**Affiliations:** 1 Department of General Surgery, Huashan Hospital and Cancer Metastasis Institute and Institutes of Biomedical Sciences, Fudan University, Shanghai, 200032 China; 2 Department of General, Visceral and Cancer Surgery, University Hospital of Cologne, Cologne, Germany

**Correction:** ***J Exp Clin Cancer Res*** **37, 179 (2018)**

10.1186/s13046-018-0832-1

Following publication of the original article [1], errors were found in Fig. 5a due to misused of images in Hep3B-SCR/EV, -shOPN/EV, and -shOPN/DNMT1.

The correct figure is given below:

**Fig. 5:**
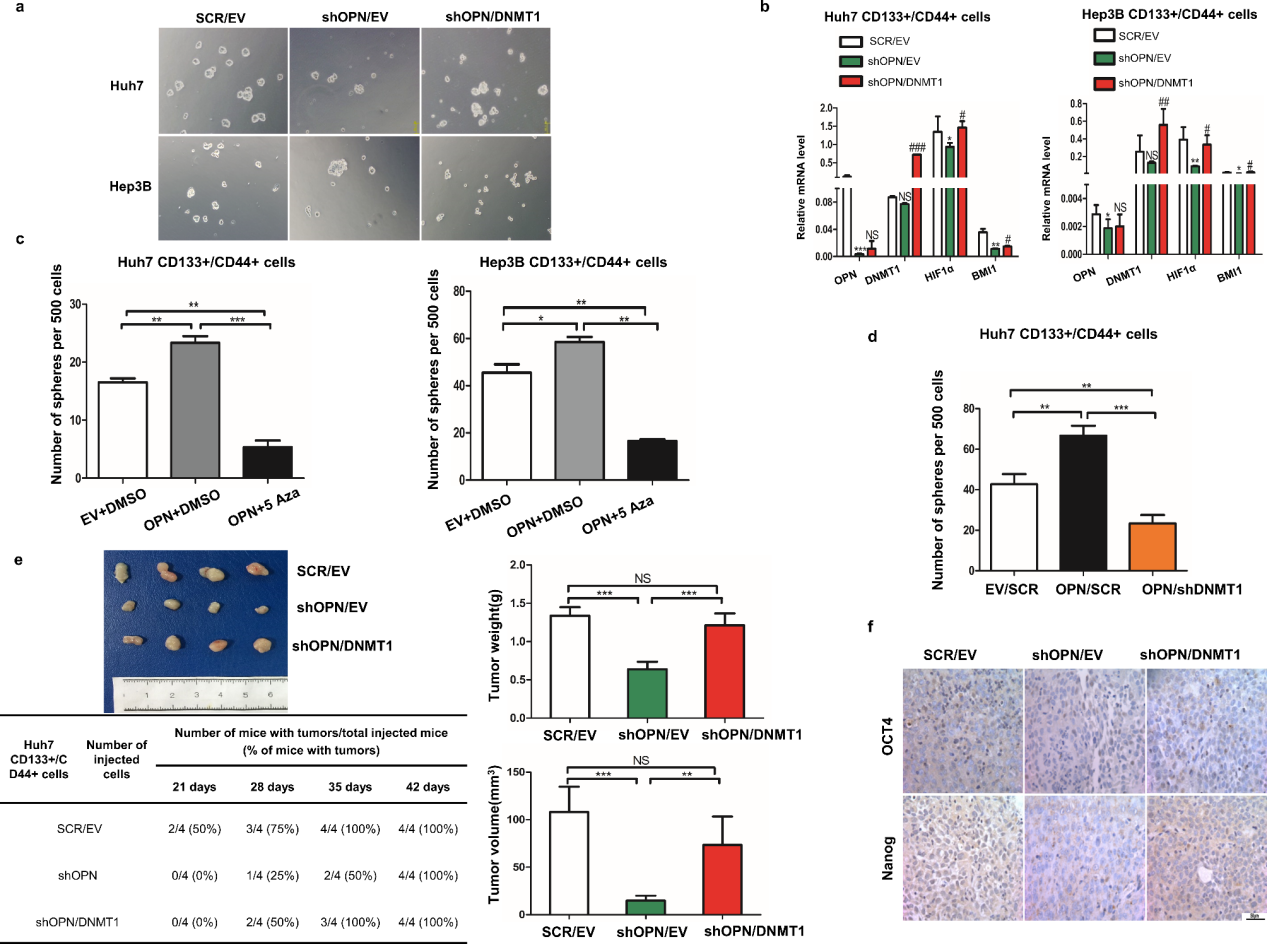
OPN modulates the properties of CD133+/CD44 + cells through regulating DNMT1. **a** When CD133+/CD44 + cells with shOPN were transfected with DNMT1 from Huh7 and Hep3B cells, the ability of sphere forming was rescued compared to the shOPN group, 40X. **b** The expression of indicated genes down regulated in CD133+/CD44 + cells from Huh7 and Hep3B transfected with shOPN compared to the control, were reversed in part by DNMT1 transfection. **c** The number of spheres formed by CD133+/CD44 + cells with EV, CD133+/CD44 + cells with OPN and CD133+/CD44 + cells with OPN treated by 5 Aza, **, *p* < 0.01, ***, *p* < 0.001. **d** The number of spheres formed by CD133+/CD44 + cells with EV/SCR, CD133+/CD44 + cells with OPN/SCR and CD133+/CD44 + cells with OPN/shDNMT1, **, *p* < 0.01. **e** CD133+/CD44 + SCR/EV, CD133+/CD44 + shOPN/EV and CD133+/CD44 + cells shOPN/DNMT1 from Huh7 were injected subcutaneously into the mice, and recorded the time of tumor initiation, tumor weight and tumor volume, **, *p* < 0.01, ***, *p* < 0.001, NS, no significance. **f** IHC staining of stemness-related markers, OCT4 and Nanog in the tumor tissues derived from CD133+/CD44 + cells with SCR/EV or shOPN/EV or shOPN/DNMT1
