# Supplementary material for: Osteopontin alters DNA methylation through up-regulating DNMT1 and sensitizes CD133+/CD44+ cancer stem cells to 5 azacytidine in hepatocellular carcinoma
Source: J Exp Clin Cancer Res. 2018 Jul 31;37:179. doi: 10.1186/s13046-018-0832-1 (PMC6069805; doi:10.1186/s13046-018-0832-1)
Supplement: Supplementary file 1 — Supplemental materials and methods. (DOCX 16 kb) [file 13046_2018_832_MOESM1_ESM.docx]

**Supplemental materials and methods**

**Plasmids construction and transfection**

Over-expressed lentivirus plasmids were constructed with specific primers, DNMT1 forward primer-5’-TGAAGCCTCCGAGATGCCGGC-3’, reward primer-5’- AGGAGGAAGCTGCTAAGGACTAG -3’. The expression plasmid for intracellular form of OPN deleting the codons from 1 to 15 was generated from full length OPN expression plasmid by PCR-mediated mutagenesis. Full length OPN plasmid and shOPN were constructed as previously described, as well as methods of cell transfections (1).

**Western blot assay**

Whole cell lysis was produced by using RIPA buffer containing protease inhibitor. Proteins were separated by sodium dodecyl sulfate polyacrylamide gel electrophoresis (SDS-PAGE) and transferred to polyvinylidene fluoride (PVDF) membranes. After blocking with 5% non-fat milk, membranes were incubated with the primary antibody and secondary antibody. The following antibodies were used: anti-DNMT1 (1:1000, Active Motif, 39204), anti-OPN (1:1000, Abcam, ab69498), anti-OCT4 (1:1000, Abcam, ab137427), anti-CD133(1:1000, Rockland Immunochemicals, 600-401-DT2), anti-N-Cad (1:1000, CST, 4061S), anti-β-actin (1:2000, Proteintech, HRP-60008), and goat-anti-rabbit/mouse IgG conjugated to horseradish peroxidase (HRP) (1:5000, Cwbiotech, CW0102S and CW0103S). And each band was detected with Image Acquisition using ImageQuant™ LAS 4000 (GE Healthcare Life Sciences).

**RT-PCR**

RNA was extracted from cells and frozen samples by using trizol (Invitrogen, California, USA) and then reversely transcripted into cDNA. Real-time PCR was performed using SYBR Green PCR Master Mix (DBI Bioscience, Denmark) and ABI PRISM 7900 Sequence Detection System (Applied Biosystems). Results were normalized to β-actin for mRNA measurement.

**Flow cytometry**

2×10^6^ cells were stained with the antibodies for 15min at room temperature and washed by PBS for one time. Cells were resuspended with 300ul PBS and analyzed by flow cytometry (Epics Altra, Coulter, USA).

**Sphere forming assay**

Sphere formation was conducted by seeding 500 cells per well into 6-well or 24-well ultra-low attachment plate (Corning Incorporated Life Sciences, Acton, MA) in serum-free DMEM/F12 medium (Gibco), B27 (50×, Invitrogen), N2 (100×, Invitrogen), 20 ng/ml bFGF, 10 ng/ml EGF (Pepro-tech, Rocky Hill, NJ) as additives. Cells were incubated in a 5% CO_2_ humid incubator for one week and spheres were imaged to counter under a stereomicroscope (Olympus, Shinjuku-ku, Tokyo, Japan). This assay was repeated for triple times.

**Dot blot assay**

Genomic DNA was extracted by using phenol-chloroform and diluted into the same concentration. Then denatured the DNA with 10mM NaOH for 15min in 98℃ and added 1μl of each sample into nitrocellulose filter membrane. After drying thoroughly, apply UV for crosslink. Be blocked with 5% non-fat milk and incubated with primary antibodies and second antibodies. Finally, 5-mC was detected with Image Acquisition using ImageQuant™ LAS 4000 (GE Healthcare Life science).

**Methylation-Specific PCR, MSP**

Genomic DNA was isolated from the cells and performed bisulfite conversion (Methylation-Gold Kit, ZYMO). And then use PCR with two pairs of primers that selectively amplified either methylated or unmethylated DNA. Run the PCR products on 2% agarose gel.

**Reference**

1. Sun BS, Dong QZ, Ye QH, Sun HJ, Jia HL, Zhu XQ, et al. Lentiviral-mediated miRNA against osteopontin suppresses tumor growth and metastasis of human hepatocellular carcinoma. HEPATOLOGY. 2008 2008-12-01;48(6):1834-42.
