# Supplementary material for: Osteopontin alters DNA methylation through up-regulating DNMT1 and sensitizes CD133+/CD44+ cancer stem cells to 5 azacytidine in hepatocellular carcinoma
Source: J Exp Clin Cancer Res. 2018 Jul 31;37:179. doi: 10.1186/s13046-018-0832-1 (PMC6069805; doi:10.1186/s13046-018-0832-1)
Supplement: Supplementary file 2 — Figure S1. The expression of OPN and DNMT1. (A) qRT-PCR and immunoblot assay of OPN knockdown. (B) qRT-PCR assay of DNMT1 knockdown. Figure S2. OPN knockdown impaired the properties of CD133+/CD44+ cells. (A-B) OPN rescuing reversed the number of spheres and the expression of genes in Huh7, NS, no significance. (C) shOPN in CD133+/CD44+ cells from Hep3B reduced the number and size of spheres, 100x. (D) CD133 and CD44 were down-regulated in CD133+/CD44+ shOPN from Hep3B. (E) OPN knockdown in CD133+/CD44+ cells from Hep3B inhibited genes expression. (F) shOPN in Hep3B CD133+/CD44+ cells decreased the potential of migration on gelatin, 100x and 400x. (G-H) OPN rescuing reversed the number of spheres and the expression of genes in Hep3B. Figure S3. OPN strengthened the stemness of CD133+/CD44+ cells from Huh7. (A-C) OPN over-expression formed more spheres of larger size, 100x, and activated genes expression. (D) Mice injected with 1,000 cells of CD133+/CD44+ EV or OPN were monitored weight and volume of tumors. Figure S4. MeDIP-seq results of RASSF1, CDKL2 and GATA4. Figure S5. Statistical analysis of iTRAQ assay. (A) KEGG analyses in Huh7 CD133+/CD44+ cells with SCR or shOPN. (B) Signaling pathways analyses. Figure S6. DNMT1 rescued the potential of sphere formation of CD133+/CD44+ cells with shOPN. (A)The number of spheres formed by CD133+/CD44+ cells with SCR/EV, shOPN/EV or shOPN/DNMT1. Figure S7. OPN related to DNMT1 expression. (A) The expression of DNMT1-downstream genes in CSCs with SCR or shOPN. (B) Staining of E-cadherin and GATA4 in the tumor formed by CSCs with SCR or shOPN. (C) The correlation of OPN and DNMT1 in tumor tissues (data form TCGA). Figure S8. CD133+/CD44+ cells with low OPN showed less sensitivity to 5 Aza. (A) 5 Aza IC50 (μM) in CD133+/CD44+ cells with SCR or shOPN. (B) Staining of OPN in the patient tissues. (DOCX 2324 kb) [file 13046_2018_832_MOESM2_ESM.docx]

Figure S1

**
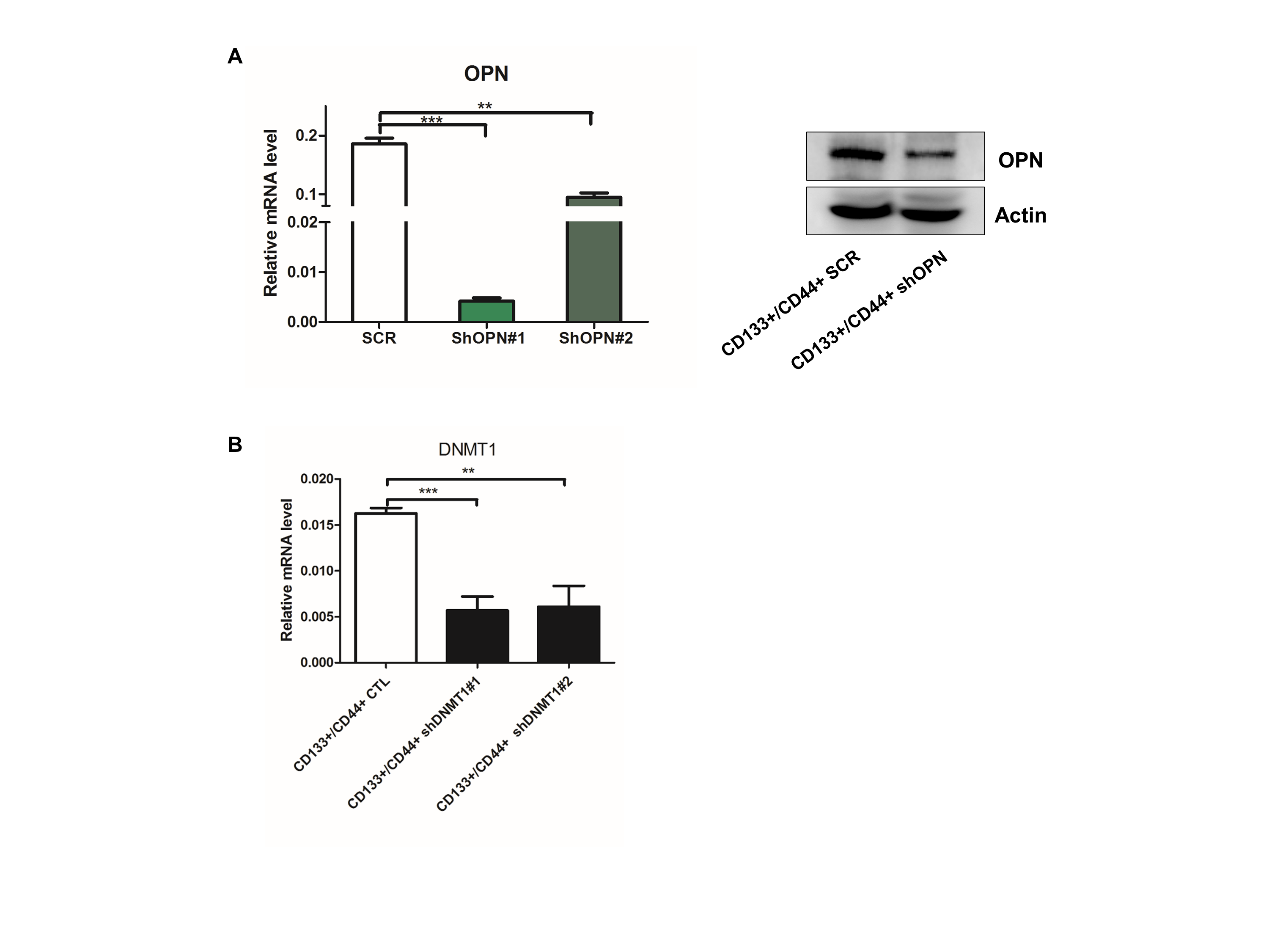
**

**Figure S1.** (**A**) qRT-PCR and immunoblot assay of OPN knockdown. (**B**) qRT-PCR assay of DNMT1 knockdown.

Figure S2

**
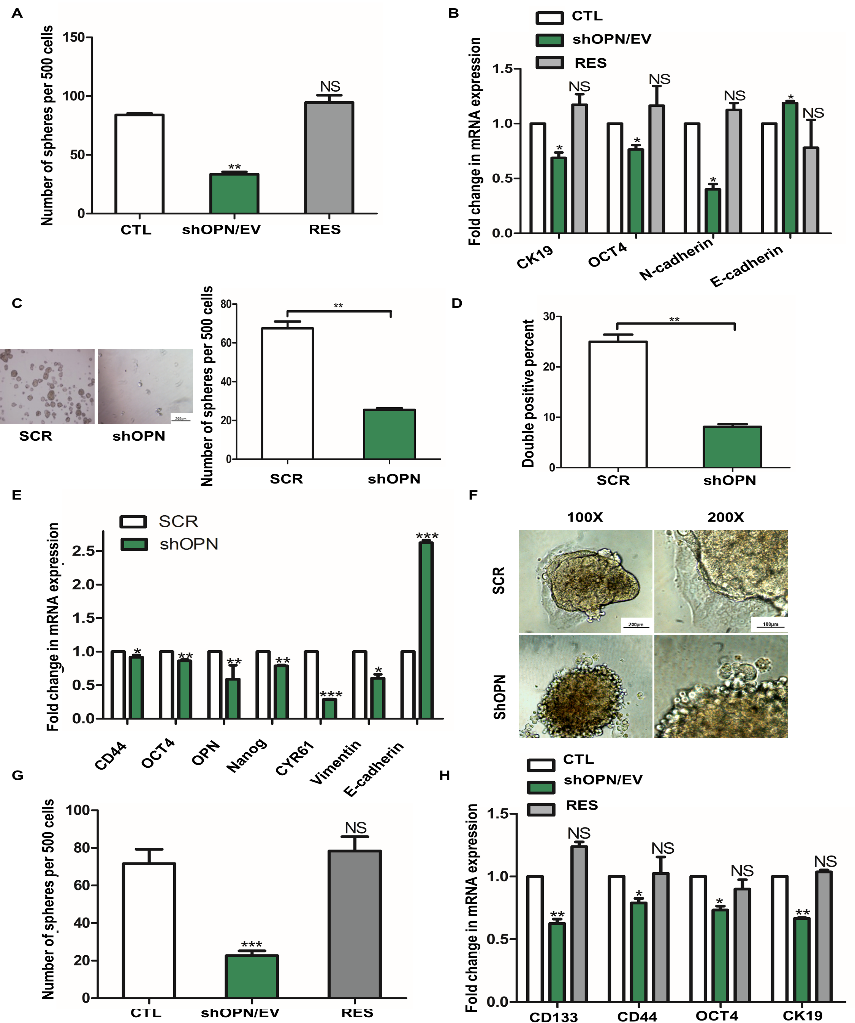
**

**Figure S2. knockdown of OPN impaired the properties of CD133+/CD44+ cells.** (**A**-**B**) When OPN was put back to the OPN knockdown stable cells from Huh7, the number of spheres was almost the same as the control and the genes down-regulated in CD133+/CD44+ shOPN/EV, were expressed no significant differently compared with these in CD133+/CD44+ CTL, *, *p*< 0.05, NS, no significance. (**C**) shOPN in CD133+/CD44+ cells from Hep3B reduced the number and size of the spheres significantly, 100x, **, *p*< 0.01. (**D**) Flow cytometry analysis showed CD133 and CD44 were both down-regulated in CD133+/CD44+ shOPN from Hep3B, **, *p*< 0.01. (**E**) knocking down OPN in CD133+/CD44+ cells from Hep3B inhibited the stemness-related genes expression compared with the control. (**F**) shOPN in Hep3B CD133+/CD44+ cells decreased the potential of migration on gelatin, 100x and 400x. (**G**-**H**) When OPN was put back to the OPN knockdown stable cells from Hep3B, the number of spheres was almost the same as the control and the genes down-regulated in CD133+/CD44+ shOPN/EV, were expressed no significant differently compared with these in CD133+/CD44+ CTL, *, *p*< 0.05, NS, no significance.

Figure S3


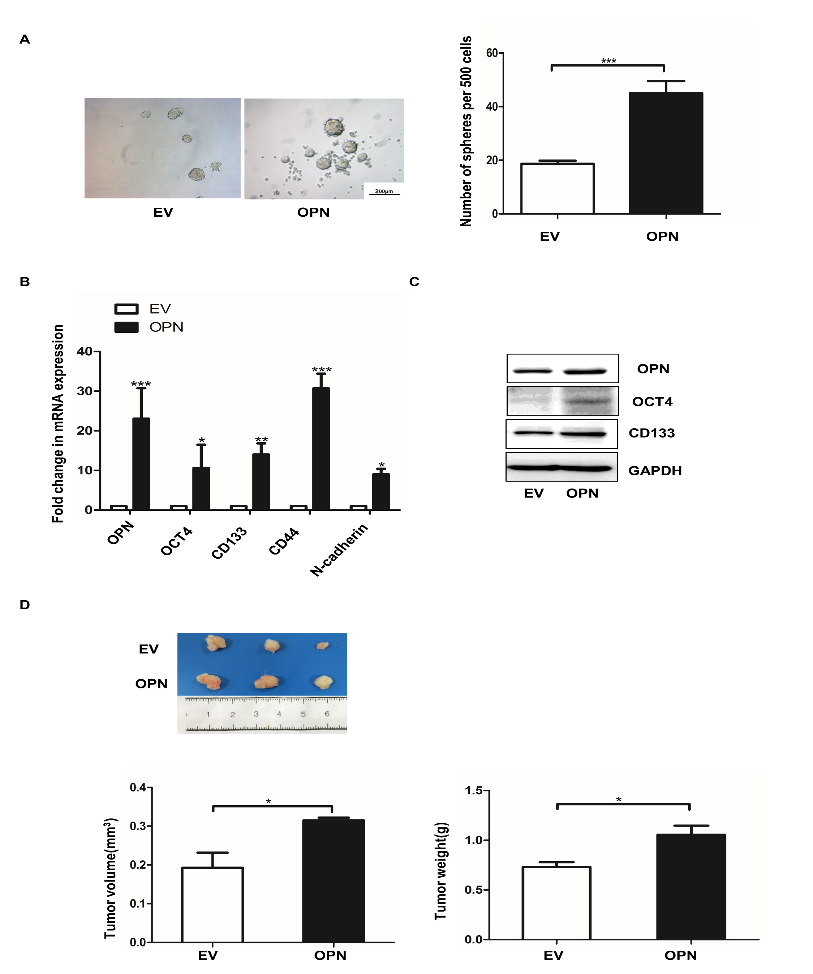


**Figure S3. Over expression of OPN strengthened the stemness of CD133+/CD44+ cells from Huh7.** (**A**) CD133+/CD44+ cells sorting from Huh7 with OPN over expressed formed more spheres of larger size than the control, 100x, ***, *p*< 0.001. (**B**-**C**) CD133+/CD44+ cells with stably transfected with OPN activated some genes expression by western blot and qRT-PCR, *, *p*< 0.05, **, *p*< 0.01 and ***, *p*< 0.001. (**D**) In vivo, 1,000 cells of CD133+/CD44+ EV and CD133+/CD44+ OPN were subcutaneously injected into NOD SCID mice, monitored the weight and volume of the tumor, *, *p*< 0.05.

Figure S4


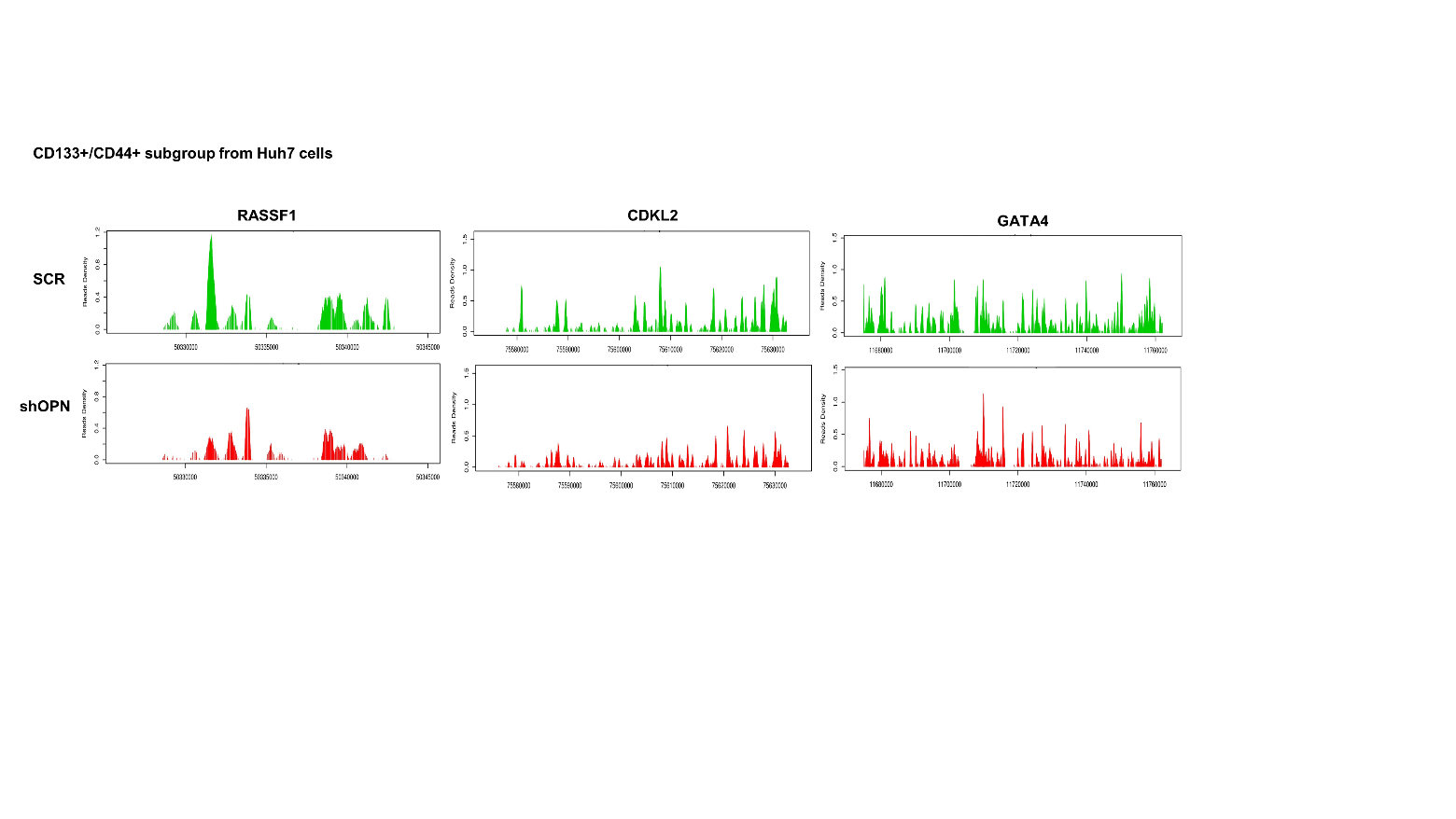


**Figure S4. MeDIP-seq results of *RASSF1*, *CDKL2* and *GATA4* genes.**

Figure S5


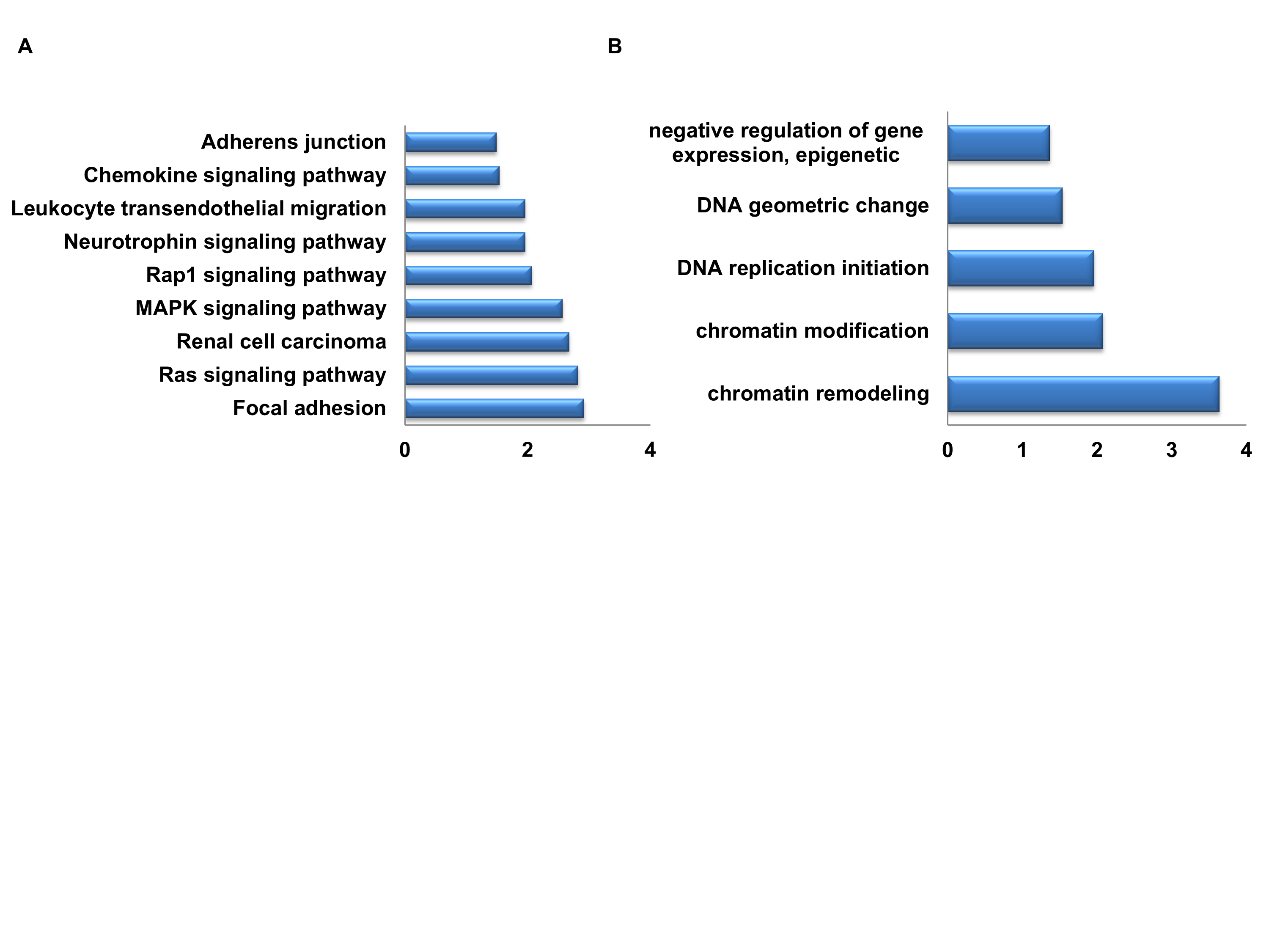


**Figure S5. Statistical analysis of iTRAQ assay.** (**A**) KEGG analyses of iTRAQ assay in Huh7 CD133+/CD44+ cells with SCR or shOPN. (**B**) some signaling pathways related to chromosome stability and regulating gene expression epigenetically.

Figure S6


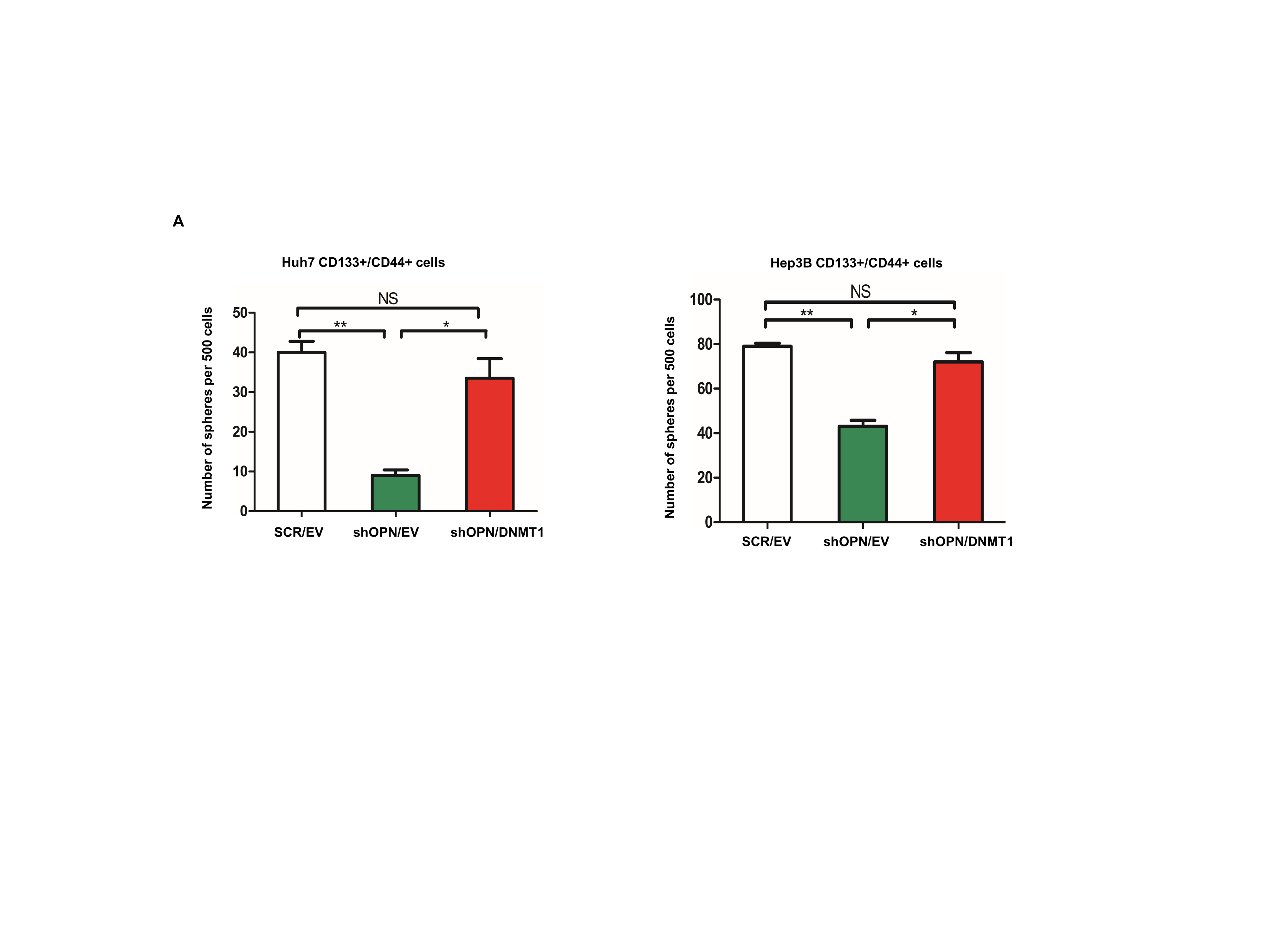


**Figure S6. DNMT1 introduction rescued the potential of sphere formation of CD133+/CD44+ cells with shOPN.** (**A**)The number of spheres formed by CD133+/CD44+ cells with SCR/EV, CD133+/CD44+ cells with shOPN/EV and CD133+/CD44+ cells with shOPN/DNMT1.

Figure S7

**
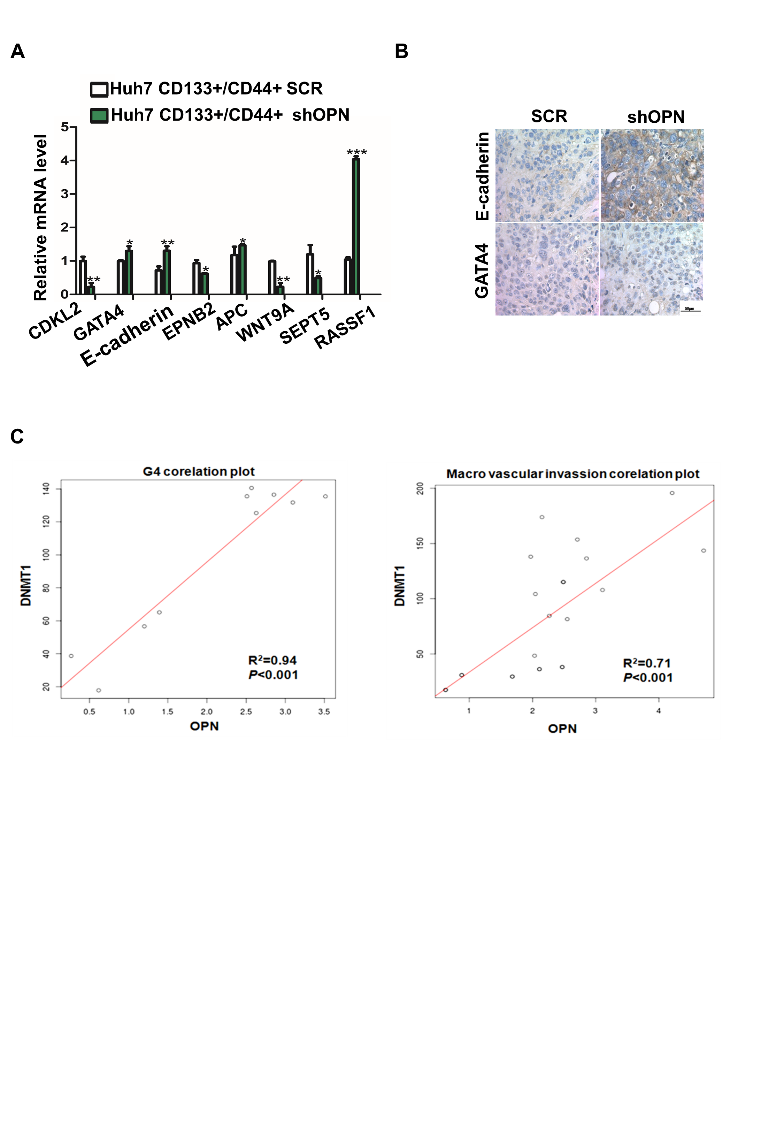
**

**Figure S7. OPN related to DNMT1 expression**. (**A**) The expression level of DNMT1-downstream genes in CSCs with SCR or shOPN. (**B**) IHC staining of E-cadherin and GATA4 in the tumor tissues formed by CSCs with SCR or shOPN. (**C**) The correlation of OPN and DNMT1 in tumor tissues (data form TCGA).

Figure S8


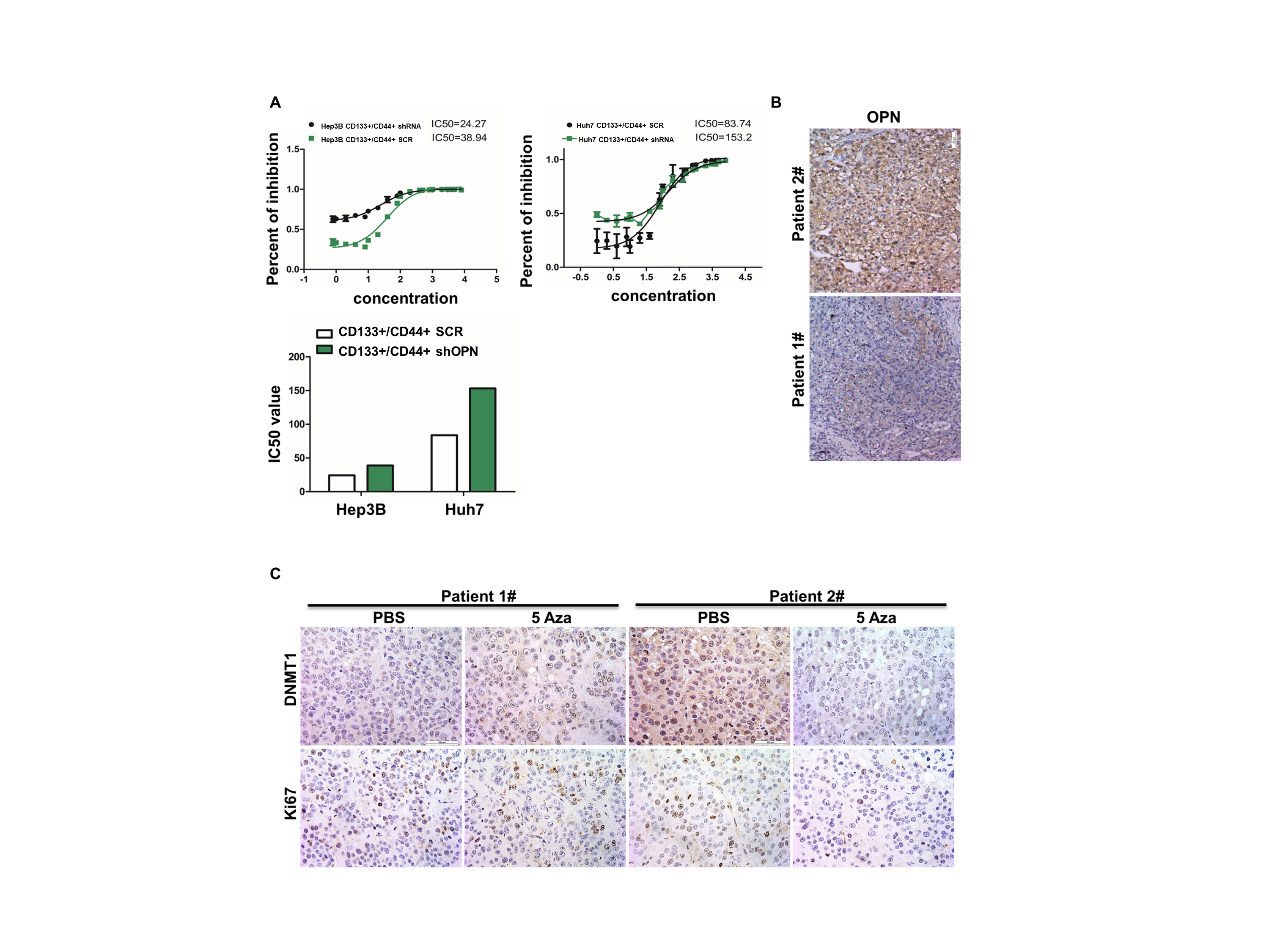


**Figure S8. CD133+/CD44+ cells with low OPN showed less sensitivity to 5 Aza.** (**A**) 5 Aza IC_50_ (μM) in CD133+/CD44+ cells with SCR or shOPN from Huh7 and Hep3B. (**B**) IHC staining of OPN in the tissues from two HCC patients.
